# A Pilot Study on Across-Generation Impacts of Maternal Heat Stress on Blood Metabolites of Female Holstein Dairy Calves

**DOI:** 10.3390/metabo13040494

**Published:** 2023-03-29

**Authors:** Kathrin Halli, Imke Cohrs, Kerstin Brügemann, Christian Koch, Sven König

**Affiliations:** 1 Institute of Animal Breeding and Genetics, Justus-Liebig-University Gießen, Ludwigstraße 21 b, 35390 Gießen, Germany; 2 Educational and Research Centre for Animal Husbandry, Hofgut Neumuehle, 67728 Muenchweiler an der Alsenz, Germany

**Keywords:** dairy calves, maternal heat stress, plasma metabolome

## Abstract

Heat stress (HS) during late gestation implies unfavorable effects on dairy cows and their in-utero heat stressed offspring. The objective of the present study was to elucidate the effect of intrauterine (maternal) HS during the last week of gestation on blood metabolite concentrations of female dairy calves during their first week of life. We defined the mean temperature humidity index (mTHI) during the last gestation week of ≥60 as threshold for maternal HS. In this regard, we compared differences in metabolite concentrations of maternally heat stressed (MHS_CALVES_) (n = 14) and not heat stressed (NMHS_CALVES_) (n = 33) calves. We identified 15 metabolites from five different biochemical classes (phosphatidylcholines, cholesteryl esters, sphingomyelins, cresols and hexoses) as potential biomarkers for maternal HS in calves. The plasma concentrations of all significantly affected metabolites were lower in MHS_CALVES_ when compared to NMHS_CALVES_. The effect of maternal HS during the last week of gestation on blood metabolite concentrations of the female offspring during the first week after birth might be due to HS induced intergenerational physiological alterations, impaired colostrum quality or epigenetic modifications of the calf genome. The results of this pilot study should be validated in ongoing fully standardized studies.

## 1. Introduction

The detrimental effects of heat stress (HS) during late gestation on the mature multiparous cow with severe consequences for several physiological mechanisms and behavioral traits in the following lactation are well known (e.g., [1,2]). Despite their suggested higher heat tolerance when compared to multiparous dry cows, quite similar HS effects during late gestation on production traits were detected for nulliparous heifers [3]. Due to the lower metabolic heat production, dry cows respond less sensitive to HS than lactating dairy cows. However, modifications of the endocrine system of dry cows during moderate HS were observed [4]. Prompt HS also affects calves, but to a lesser extent than for lactating dairy cows. Nevertheless, also calves suffer from HS to some degree, with detrimental effects on dry matter intake (DMI) and on growth performance [5]. From an across-generation perspective, several recent publications addressed the effects of in-utero (maternal) HS during late gestation in cows and heifers on growth, immune function, thermotolerance, metabolism and performance traits of their calves (e.g., [6,7,8]). Skibiel et al. [9,10] found alterations in the mammary gland morphology and the methylation profile of mammary gland DNA in heifers, which suffered from fetal hyperthermia. In the same heifer group, morphology and DNA methylation changes of key metabolic organs, such as the liver, were observed [9]. Such results indicate potential HS induced epigenetic alterations of the calf’s genome during fetal development (i.e., fetal programming), inducing alterations of postnatal phenotypes [6]. Laporta et al. [6] found impaired daughter survival and milk production in parities 1, 2 and 3 due to maternal heat stress during late gestation. With regard to economy, Laporta et al. [6] calculated an annual economic loss of 595 million dollars for the United States dairy sector including additional costs for rearing heifers, shorter productive cow life and milk yield decline in the F1 generation. 

With regard to an altered metabolism, maternally heat stressed dairy calves showed higher plasma insulin concentration at day 1 after birth [11], and a faster glucose clearance during a glucose tolerance test and an insulin challenge [12]. Dado-Senn et al. [13] postulated an altered metabolism, inducing reduced feeding motivation in prenatally heat stressed calves. 

The analysis of blood biochemical parameters reflecting the metabolic state of maternally heat stressed calves might contribute to the detection of potential HS biomarkers. A variety of blood [14], milk [15] and liver [16] metabolites already serve as potential biomarkers for HS effects in dairy cows. In cattle, Monteiro et al. [17] found associations between maternal HS during late gestation and alterations of postnatal blood metabolite profiles of offspring. Specifically, concentrations of nonesterified fatty acids at the age of 32 days were higher in the HS group compared to calves of the cooled dam control group. Explanations address an altered preference of energy source in calves which suffered from intrauterine HS, because they prefer to use glucose instead of fatty acids or ketone bodies.

The aim of the present study was to infer the effects of intrauterine (maternal) HS during the last gestation week on blood plasma metabolites of female dairy calves. We hypothesize significant differences in concentrations of individual plasma metabolites between maternally heat stressed calves (MHS_CALVES_) and not maternally heat stressed calves (NMHS_CALVES_), suggesting metabolites as biomarkers for time-lagged HS effects. 

## 2. Materials and Methods

### 2.1. Animals, Housing System, Feeding and Management

All experimental procedures that were performed in this study are approved by the local authority for animal welfare affairs (Landesuntersuchungsamt Rheinland-Pfalz, Koblenz, Germany), and are in accordance with the German Animal Welfare Act (permit number: A19-20-002 EV).

The study included a total number of 60 female German Holstein dairy calves, born during a 12 months period from February 2020 to February 2021 at the Educational and Research Centre for Animal Husbandry, Hofgut Neumühle in Münchweiler a. d. Alsenz, Germany. All cows (i.e., the dams of the calves in this study) were kept in a freestall dairy shed with same housing characteristics across seasons. Cows were from parities one to five, were inseminated by artificial insemination and were dried off seven weeks before the expected calving date. All cows were fed the same total mixed ration (TMR) ad libitum diet during the experiment in the dry period. The TMR was prepared in the morning and delivered twice per day. 

All calves received an identical colostrum and milk replacer program. Utilization of the calving sensor system Moocall (Moocall LTD, Bluebell, Dublin, Ireland) ensured a colostrum intake of 3 L from the respective dam within the first 2 h after birth. For calves not drinking independently, the same amount of colostrum was drenched. From birth until day 10 of life, all calves were kept in straw-bedded single calf hutches. The feed supply included fresh water ad libitum and small amounts of hay. Until the age of 5 days, calves received 6 L of their dam’s transition milk twice per day. Afterwards (from the 10th meal onwards until the age of 14 days), calves received high quality milk replacer (2 × 6 L/d). All female calves were blood sampled during the first age week. We excluded blood samples if blood sample preparation for metabolic phenotyping failed the standard protocol guidelines, e.g., due to technical problems. The final dataset included 47 female calves. 

### 2.2. Climate Data Recording and HS Index

Climate data in the “dry cow environment” were recorded using electronic data loggers (Tinytag Plus 2 TGP-4500, Gemini Data Loggers). Loggers were installed in the dairy shed above the calving pens and recorded temperature and humidity in intervals of one hour. Climate data was used to calculate a temperature humidity index (THI) using the formula of the National Research Council (NRC) [18]:

THI = [(1.8 × T (°C)) + 32] [0.55 − (0.0055 × RH (%))] × [(1.8 × T (°C)) − 26],(1)

where T is the dry bulb temperature and RH is the relative humidity. In a next step, the average THI per day was used to calculate a weekly mean THI (mTHI). The mTHI, calculated for the week before birth, was classified into two different classes: class 1 (no HS): mTHI < 60, and class 2 (HS): mTHI ≥ 60. The definition of the HS-threshold THI ≥ 60 was based on the results by Brügemann et al. [19], who evaluated the THI formula for Holstein dairy cows under German climatic conditions. Due to missing HS experiences in calves, THI 60 was the chosen threshold for calf group creation. Accordingly, calves were allocated either to the group which suffered from maternal HS during the last week of gestation (MHS_CALVES_; n = 14), or to the group without maternal HS (NMHS_CALVES_; n = 33). Our decision to focus on the last week of gestation based on the findings of previous studies of our working group (e.g., [20,21,22]), in which we identified an important effect of maternal HS during the last week of gestation on conventional performance traits and health diagnoses of calves. 

### 2.3. Blood Sampling and Blood Plasma Metabolome Profiling

Blood samples from calves were collected once between day one and day seven after birth and between 10:00am and 15:15pm using EDTA tubes (S-Monovette, Sarstedt AG & Co, Nürnberg, Germany). According to the guidelines for blood sample preparation for metabolic phenotyping (Biocrates Life Science AG, Innsbruck, Austria), cells and plasma were separated by centrifugation (2500× *g*, 10 min, 20–24 °C) immediately after sample collection. Directly after centrifugation, blood plasma was frozen at −80 °C. Metabolome analysis was carried out using a targeted quantitative metabolomics approach employing the MxP Quant 500 Kit (Biocrates Life Science AG), which identifies and quantifies 630 different metabolites of 26 biochemical classes (alkaloids (1), amine oxides (1), amino acids (20), amino acid related (30), bile acids (14), biogenic amines (9), carbohydrates and related (1) (hexoses—90 to 95% glucose), carboxylic acids (7), cresols (1), fatty acids (12), hormones and related (4), indoles and derivatives (4), nucleobases and related (2), vitamins and cofactors (1), acylcarnitines (40), lysophosphatidylcholines (14), phosphatidylcholines (76), sphingomyelins (15), ceramides (28), dihydroceramides (8), hexosylceramides (19), dihexosylceramides (9), trihexosylceramides (6), cholesteryl esters (22), diglycerides (44), triglycerides (242)). Concentrations (µM/L) of all metabolites were determined with mass spectrometry. Flow injection analysis-tandem mass spectrometry using a 5500 QTRAP^®^ instrument (AB SCIEX, Darmstadt, Germany) with an electrospray ionization source was used to measure lipids (12 of the 26 biochemical classes) and hexoses. Small molecule classes (14 of the 26 biochemical classes) were analyzed by liquid chromatography mass spectrometry. The experimental metabolomics measurement technique is described in detail in the patents EP1897014B1 [23] and EP1875401B1 [24]. 

### 2.4. Data Analysis and Visualization

In a first step, blood plasma metabolome data were analyzed using the web-based platform MetaboAnalyst V5.0 [25]. Regarding outlier data, we excluded metabolites if ≥50% of the respective measurements were below the limit of detection. After exclusion of 371 metabolites, the metabolite dataset of calves included 259 metabolites of 22 biochemical classes. Furthermore, metabolite data were normalized by log transformation and Pareto scaling [26]. In a standard cross-sectional 2-group study, we compared differences between metabolite concentrations of MHS_CALVES_ and NMHS_CALVES_. Differences were first assessed by volcano plots (false discovery rate adjusted *p* < 0.05), whereby important metabolites were identified based on a fold change threshold of two on the x-axis and t-test threshold (*p*-value) of 0.1 on the y-axis. Afterwards, we implemented a partial least squares-discriminant analysis (PLS-DA). Accordingly, the data were categorized into a few latent variables maximizing the covariance between the response and the predictors. A VIP (variable importance in the projection) plot ranked the 15 metabolites based on their importance in discriminating the HS-animals from the NHS-animals. With regard to group discrimination, the most important metabolites were identified according to the highest VIP values, with VIP values > 1 denoted as significant and VIP values >2 denoted as highly significant. In order to validate the PLS-DA, we implemented a permutation testing with 2000 random resamplings. The test procedure determines whether the separation between the HS- and NHS-groups are still a result of chance (if *p* > 0.05). In addition, a sparse partial least squares-discriminant analysis (sPLS-DA) was used to identify the 15 compounds mostly contributing for the variation between HS- and NHS-animals. Lastly, we visualized the differences in metabolite concentrations between the different groups via heatmap applications. In a second step, the important blood metabolites identified via MetaboAnalyst V5.0 were further analyzed using linear mixed models as implemented in the MIXED procedure of SAS University Edition (SAS Institute, Cary, NC). The basic statistical model included the fixed effect ‘mTHI class’, which was stepwisely supplemented for each metabolite by the fixed effects ‘age of calf at blood sampling (days), ‘calving condition’, ‘birth weight class for calves’ and ‘time of blood sampling’. For each metabolite, the Akaike information criterion (AIC) was the evaluation criterion to identify the best statistical model. The levels for fixed effect classes were:

- age of calf: blood sampling at 1st day of age = class 1; blood sampling at 2nd to 3rd day of age = class 2; blood sampling at 4th to 5th day of age = class 3; blood sampling at 6th to 7th day of age = class 4
- calving condition: no birth assistance = class 1; minor birth assistance or dystocia = class 2
- birth weight class for calves: birth weight from 34.8 kg to 39.0 kg = weight class 1; birth weight from 39.1 kg to 48.0 kg = weight class 2
- time of blood sampling: blood sampling from 10:00 a.m. to 12:00 p.m. (morning hours) = class 1; blood sampling from 12:01 p.m. to 15:15 p.m. (afternoon hours) = class 2

The statistical linear mixed models as applied according to the lowest AIC-value for the individual metabolites are described in the following Table 1. 

For all models, the threshold for statistical significance was *p* < 0.05. *p*-values were corrected for multiple testing using the Bonferroni correction. Relationships among metabolite concentrations and calf birth weight were assessed by calculated Pearson’s correlation coefficients. 

## 3. Results

### 3.1. Climate Data

**Figure 1:**
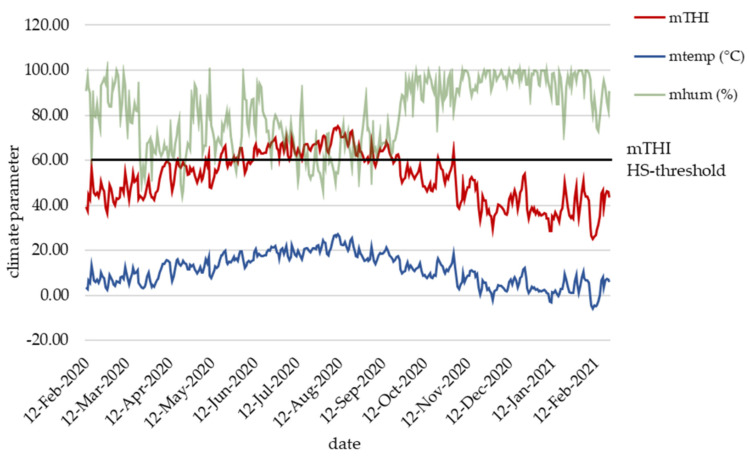
Daily mean temperature humidity index (THI), daily mean temperature (temp) and daily mean humidity (hum) during the recording period from February 2020 to February 2021.

During the complete recording period, the daily mean THI ranged between 25 and 75 (Figure 1). Heat stress days (daily mean THI ≥ 60) were frequently observed from May to September 2020. Three further single-day HS events occurred on 18th of April, 22nd of October and on 2nd of November 2020. Two longer lasting HS periods with THI persistently above the were observed from 11th of June to 29th of August 2020, and from 8th to the 19th of September 2020. From February to April 2020 and from October 2020 to February 2021, cows and calves did not suffer from HS. All dams calved throughout the year, indicating that calves born from May to September 2020 had the highest risk to suffer from maternal HS.

### 3.2. Animal Performance

The birthweight of the 47 female calves ranged from 34.8 kg to 48.0 kg. Detailed information about individual mTHI classification, age at blood sampling (d), time of blood sampling, birthweight (kg) and calving condition of calves is provided in Table 2. A complete table with raw metabolite concentrations of all 259 metabolites is provided in Supplementary Table S1.

There was no significant difference between dam groups for MHS_CALVES_ and NMHS_CALVES_ with regard to (mean ± SEM) parity, weight and milk yield of multiparous cows when dried off or with regard to weight of primiparous cows before calving (Table 3). Therefore, we did not include these effects in the model.

### 3.3. Phenotypic Correlations between Metabolite Concentrations and Birth Weight in Calves

Phenotypic correlations between metabolite concentrations (µM) and birth weight with their respecitve *p*-values for the test of significant devition from zero are shown in Table 4. 

Phenotypic correlations between metabolite concentrations and birth weight of calves ranged from −0.08 to 0.26. Except for p-Cresol sulfate (p-Cresol-SO4), correlations were generally positive. However, no significant correlations between the concentration of metabolites and birthweight of calves were found. 

### 3.4. Effect of Maternal HS during the Last Week before Birth on Blood Metabolite Profiles of Calves

**Figure 2:**
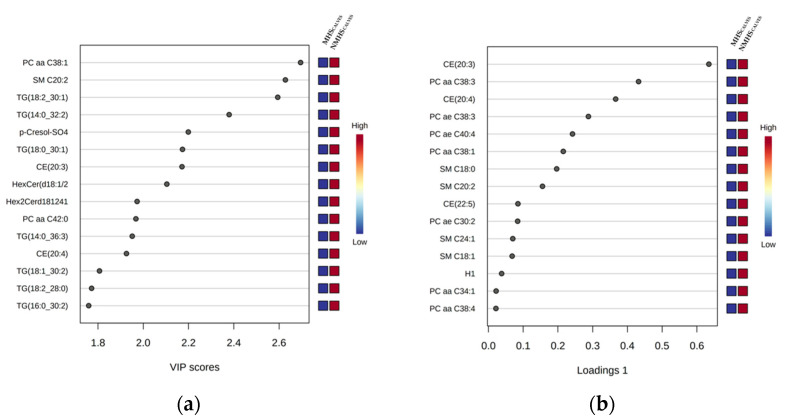

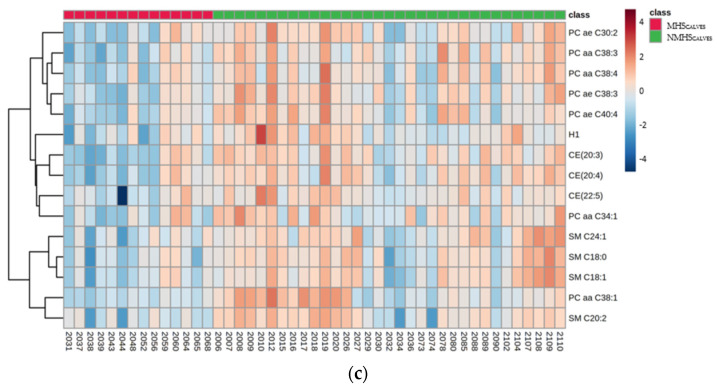
(**a**) VIP (variable importance in the projection) scores, calculated via PLS-DA (partial least squares-discriminant analysis), ranking the compounds listed on the left. The rank of the compounds correlates with the contribution of the compound to the overall variation represented by the first component (PC1) between MHS_CALVES_ and NMHS_CALVES_. Only the 15 metabolites with the highest VIP scores are shown. (**b**) Loadings plot (Loadings of component 1) as calculated by sPLS-DA, where the metabolites on the left are ranked according to the absolute values of their loadings. The relative difference of the metabolites between MHS_CALVES_ and NMHS_CALVES_ are displayed by the color scheme on the right. (**c**) Heatmap visualizing differences in metabolite concentrations of the 15 most interesting metabolites of MHS_CALVES_ and NMHS_CALVES_ where each column represents the metabolite profile of one calf.

The volcano plot analyses identified significant differences in metabolite concentrations between both groups (MHS_CALVES_ and NMHS_CALVES_) for sphingomyelin (SM) SM C20:2 and phosphatidylcholine (PC) diacyl (aa) C38:1 (PC aa C38:1). The permutation test of the PLS-DA revealed that the separation between the groups was still by chance (*p* > 0.05). However, the VIP plot (Figure 2a) indicates that PC aa C38:1, SM C20:2, triglyceride (TG) 18:2_30:1 (TG(18:2_30:1)), TG(14:0_32:2), p-Cresol-SO4, TG(18:0_30:1) and cholesteryl ester (CE) 20:3 (CE(20:3)) were the strongest discriminating metabolites for separating MHS_CALVES_ from NMHS_CALVES_. The color scheme on the right side showes decreased concentrations in MHSCALVES for all of the metabolites. The loadings plot of component 1 (Figure 2b), as calculated by sPLS-DA, shows the relative differences of ranked metabolites (in terms of the absolute values of their loadings) between the groups. The compounds most contrbuting to the variation between MHS- and NMHS-animals were CE(20:3), PC aa C38:3, CE(20:4), acyl-alkyl (ae) C38:3 (PC ae C38:3), PC ae C40:4, PC aa C38:1 and SM C20:2. The heatmap (Figure 2c) visualizes the 15 most interesting and differentiating metabolites of MHS_CALVES_ and NMHS_CALVES_. Results from model 1–3 indicate significant effects of the mTHI class (MHS_CALVES_ or NMHS_CALVES_) on the following 15 metabolites: PC aa C34:1 (*p* < 0.01), PC aa C38:1 (*p* < 0.01), PC aa C38:3 (*p* < 0.001), PC aa C38:4 (*p* < 0.01), PC ae C30:2 (*p* < 0.001), PC ae C38:3 (*p* < 0.001), PC ae C40:4 (*p* < 0.001), SM C18:0 (*p* < 0.001), SM C18:1 (*p* < 0.001), SM C24:1 (*p* < 0.001), CE(20:3) (*p* < 0.001), CE(20:4) (*p* < 0.001), CE(22:5) (*p* < 0.05), p-Cresol-SO4 (*p* < 0.05) and Hexoses (H1) (*p* < 0.01). A complete table with all log transformed LSMeans and corresponding standard errors of the metabolite concentrations from models 1–3 is provided in supplementary Table S2. The plasma concentrations of all significantly affected metabolites were generally lower in MHS_CALVES_ when compared to NMHS_CALVES_.

The sphingomyelin ‘SM C20:2′, the phosphatidylcholine ‘PC aa C42:0′ and the dihexosylceramide ‘Hex2Cer(d18:1/24:1)’ could not be normalized by log transformation, and consequently, these metabolites were excluded from further mixed model analyses. The fixed effect ‘calving condition’ was not significant for the plasma concentrations of PC aa C38:1 and p-Cresol-SO4. The fixed effect ‘age at blood sampling (days)’ was significant for PC ae C30:2 (*p* < 0.01), SM C18:0 (*p* < 0.001), SM C18:1 (*p* < 0.001) and SM C24:1 (*p* < 0.001), indicating increasing plasma concentrations with increasing age of calves. Hence, we observed a general calf age effect on these metabolite levels, but the calf age was not significantly different (*p* > 0.05) between both climatic groups.

## 4. Discussion

In the present study, we identified significant differences in blood metabolite concentrations between MHS- and NMHS_CALVES_ for 15 metabolites of five different biochemical classes (phosphatidylcholines, cholesteryl esters, sphingomyelins, cresols and hexoses). In addition, for four of these metabolites, we found differences in blood plasma concentrations for different ages at blood sampling with lower plasma concentrations on the days closer to birth when compared to increasing age. With regard to mTHI, the blood plasma concentrations of all significantly affected metabolites were lower in MHS_CALVES_ compared to NMHS_CALVES_. Phenotypic correlations between metabolite concentrations and birth weight of calves were slightly positive (except for p-Cresol-SO4, where the correlation was slightly negative), but not significant (*p* > 0.05). With regard to the direct HS component, small negative phenotypic correlations (−0.10) were found between birth weight and insulin-like growth factor-I in Angus beef cattle [27]. However, to our knowledge, there is no literature available addressing the phenotypic correlations between other plasma metabolites and birth weight of Holstein dairy calves. As described in the materials and methods, we tested the fixed effect ‘birth weight class for calves’ in our statistical models. However, the effect on plasma metabolite concentrations was non-significant (*p* > 0.05). The small and not significant correlations between metabolite concentrations and birth weight of calves support our findings from the mixed modelling approach.

In the following section, metabolites which were significantly affected by the mTHI class, are discussed in the context of maternal HS. Nevertheless, when interpreting our results, it should be kept in mind that the experiment in the research farm was not completely standardized, implying possible effects generated from, e.g., early gestation heat waves.

### 4.1. Lipids

Cholesteryl esters, PCs and SMs are metabolites of the class of lipids. Cholesteryl esters are formed by esterification of cholesterol with long-chain fatty acids [28]. Phosphatidylcholines are a major part of biological membranes [29], which play an important role in the lipid metabolism [30], and which are required for assembly and secretion of lipoproteins [29]. Sphingomyelins represent a class of lipids with extensive hydrogen-bonding capabilities, which is specifically enriched in the plasma membrane. Membrane SM play different roles in various cellular functions and processes [31]. However, their major functions include stabilization of cell membranes and involvement in cell signaling and apoptosis [32,33]. Sphingomyelins are synthesized by either the liver or other tissue cells or are of alimentary origin [29]. In the present study, we found significantly lower concentrations of CE(20:3), CE(20:4) and CE(22:5) (results from model 1), of PC aa C34:1, PC aa C38:1, PC aa C38:3, PC aa C38:4, PC ae C30:2, PC ae C38:3 and PC ae C40:4 (results of models 1-3) and SM C18:0, SM C18:1 and SM C24:1 (results from model 3) in MHS_CALVES_ when compared to NMHS_CALVES_. Effects of stress (e.g., HS) in cattle on such lipid classes have previously been observed. In this regard, Noble et al. [34] found significantly lower plasma CE fractions in steers exposed to heat (35 °C) when compared to steers kept at 22 °C. Tian et al. [15] found a significant downregulation of the plasma concentrations of several PC in heat stressed cows. Kenéz et al. [35] identified a decrease of SM during metabolic stress periods, especially during early lactation, suggesting deeper investigations in this regard. However, to our knowledge, no significant effect of maternal HS on plasma lipid concentrations of offspring has been documented so far. Other publications signalize a stronger impact of feeding time, feeding amount and feed composition on plasma lipid concentrations of calves. In this regard, Blum et al. [36] associated plasma cholesterol and phospholipid concentrations in calves with colostrum intake, indicating higher concentrations in calves fed colostrum immediately after birth compared to calves with delayed colostrum intake. In the study by Carroll et al. [37], plasma cholesterol levels of suckling calves fed skim milk were lower when compared to calves fed whole milk. Also, total lipid and cholesterol content of colostrum and whole milk were considerably higher when compared to skim milk. Hence, the lipid and cholesterol content in colostrum and in milk contributes to variations of the plasma cholesterol level in calves. Kenéz et al. [38] found higher PC concentrations in heifers fed milk replacer ad libitum as a calf, compared to heifers fed restricted, indicating an impact of nutrient amount in early life on calf plasma PC levels. Furthermore, they [38] found higher plasma SM concentrations (SM (OH) C14:1 and SM (OH) C16:1) in dairy calves at day 22 postpartum fed whole milk ad libitum compared to calves fed milk replacer ad libitum. The acutely absorbed components when feeding whole milk might explain the observed differences. Hence, feeding management, colostrum and milk replacer intake and its composition seem to have a marked effect on fat metabolism of calves with impact on plasma CE, PC and SM concentrations in calves. In our study, all calves received an identical colostrum and milk replacer program, and all calves received colostrum within one or two hours after birth. Hence, alterations of lipid concentrations cannot be explained by delayed colostrum feeding or lowered colostrum intake of MHS_CALVES_, but could be the result of a HS induced effect on colostrum quality of their dams. Specifically, the HS induced reduction of the fat and protein content of the colostrum might adversely affect CE, PC and SM concentrations in the plasma of calves. Such evidence was given by Almoosavi et al. [39], who reported significantly reduced colostrum protein percentage in cows suffering from HS conditions during late pregnancy. Lower colostral fat percentages were proven for heifers exposed to heat during late pregnancy [37]. Nardone et al. [40] concluded that the lower colostrum protein percentage was due to reduced blood flow toward the mammary gland with lower nutrient supply to mammary gland cells. Furthermore, they hypothesized a HS induced energy deficit to reduce the availability of fatty acid precursors, implying an impaired synthesis of short- and medium-chain fatty acids in the udder. Furthermore, we found significant effects of age at blood sampling on concentrations of SM C18:0, SM C18:1 SM C24:1 and PC ae C30:2 (results from model 3), indicating increasing plasma concentrations with increasing distance to birth. Kenéz et al. [35] found that sphingolipid concentrations of dairy cows greatly varied over time with significant differences between 5 time points before and after calving, while other compound classes remained more stable. Roelfzema et al. [41] identified increasing sphingomyelin concentrations of bovine lens increasing with age. Furthermore, a positive correlation was found between age and plasma sphingomyelin and phosphatidylcholine levels in humans, e.g., for PC ae C30:2 [42]. We hypothesize that our results are related with continuous and dynamic process of aging, including alterations of cell membrane compositions [43].

### 4.2. p-Cresol Sulfate 

p-Cresol is generated by intestinal anaerobic bacteria, such as Clostridium, Faecalbacterium, Eubacterium and others, as a degradation product of tyrosine [44]. After absorption, p-cresol is conjugated to its sulfate [45], a type of protein-bound uremic toxin [46], which induces inflammatory reactions and enhances oxidative stress [47]. In the present study, p-Cresol sulfate concentration was lower in MHS_CALVES_ compared to NMHS_CALVES_ (results from model 2). Yokoyama and Carlson [48] found ruminal Lactobacillus strains catalyzing the formation of p-Cresol. Lactobacilli appear at the first day after calf birth [49] and are the dominant bacteria in the digestive system during the first week of life [50]. The count of Lactobacillus colonies decreases with impaired colostrum quality (e.g., lower protein %) [51]. Furthermore, HS during late pregnancy was associated with reduced colostrum protein percentage in cows [39]. Hence, a HS induced reduction of colostrum protein percentage might explain the decline of Lactobacillus colonies in calves with reduced p-Cresol catalyzation.

### 4.3. Hexoses (90 to 95% Glucose)

Usually, a network including the pancreas, liver, adipose tissue, muscle and brain keeps blood glucose concentrations on a highly regulated and necessary level [52]. Nevertheless, acute HS effects on plasma glucose contents of cattle were found in a number of studies, but with conflicting results. In this regard, HS induced reductions of blood glucose concentrations were found in dairy cows [53], in six months old Holstein Friesian heifers [54], in four to five months old Holstein bull calves [55] and in Egyptian buffalo-calves [56]. Others reported of significantly increased plasma glucose content due to acute heat exposure in Egyptian buffalo calves [57] or in swamp buffaloes [58]. In contrast, maternal HS had no effects on plasma glucose levels of calves measured immediately (2 h) after birth [59], and on glucose levels measured between birth and 56 d of age [17]. However, in utero heat stressed calves had a faster glucose clearance after a glucose tolerance test before and after weaning compared to HS free calves [12,17]. In the present study, concentrations of hexoses (90 to 95% glucose in the blood of cows and other mammals [60]) were significantly lower in MHS_CALVES_ compared to NMHS_CALVES_ (results from model 1). Reasons for the discrepancies are not fully clarified. Lowered glucose levels might be due to a HS induced increase in insulin concentration, as detected in plasma samples of bull calves suffering from acute HS [55], and in serum samples of calves suffering from maternal HS during the dry period at 1 d after birth [11]. Increased insulin levels stimulate cellular glucose uptake [61], contributing to lowered glucose levels in plasma and serum. However, insulin concentrations were not analyzed in our study. Hence, we cannot proof such hypothesis.

### 4.4. Epigenetics

Effects of HS during late gestation on metabolism of calves have previously been observed [17]. In part, such effects could be attributed to epigenetics, referring to changes in genome functions caused by chemical changes in DNA and its surrounding chromatin structure [62] (e.g., altered DNA methylation [63], histone modifications [64] and microRNAs [65]). Epigenetic mechanisms further address either enhanced or repressed gene expression [62]. These changes are heritable, implying transfer of genome modifications from the parents to their offspring [66], and can be persistent through rounds of cell division [67]. Hence, late-gestation HS could alter the intrauterine environment with impact on the fetal genome due to epigenetic changes (i.e., fetal programming), resulting in different metabolic phenotypes [6]. In this regard, HS altered the methylation profile of the liver DNA in calves [9]. The liver is a key metabolic organ in lipid homeostasis, needed to satisfy the energy demands of calf growth [68]. In total, 50 genes were differentially methylated between bulls from different dam groups, i.e., heat stressed or cooled dams during late pregnancy [9]. We postulate effects of late gestation HS on metabolism of calves due to epigenetic modifications of the calf genome. Transgenerational HS effects can be studied in detail when analyzing metabolite profiles in ongoing F2 and F3 generations. In the present study, an altered metabolism in maternally heat stressed calves might cause a reduced feeding motivation [13], with negative effects on growth and performance traits. In combination with epigenetic changes in the mammary gland [9] and smaller mammary alveoli [10], HS induced metabolic changes affect the economy of the dairy industry, due to additional heifer rearing costs, a shortened productive life and a decline in milk yield [6].

## 5. Conclusions

The present study focused on an across-generational approach to analyze the effects of maternal HS during the last week of gestation on blood plasma metabolite concentrations of female dairy calves. We found significant differences in concentrations of individual plasma metabolites between calves suffering from maternal HS or not. These metabolites could be used as indicators for HS, independent from general environmental descriptors (e.g., THI). We identified 15 metabolites of five different biochemical classes (phosphatidylcholines, cholesteryl esters, sphingomyelins, cresols and hexoses) as potential biomarkers for maternal HS in calves, most relating to lipid metabolism, with overall lower metabolite levels in MHS_CALVES_. Explanations address effects of maternal HS during the last gestation week on colostrum quality as well as on epigenetic modifications, both affecting blood metabolite concentrations in calves during their first week of life. Robust metabolite biomarkers have potential (a) to develop early measures for diagnosis or even manipulation of HS induced metabolic disorders, (b) to improve the determination of the accurate threshold for the onset of HS, and (c) to improve early genetic selection for HS tolerance in dairy cows. We have to note that the prediction accuracy for HS of the identified metabolites is limited due to the missing prove of actual HS in animals. Nevertheless, this pilot study indicates first interesting physiological responses due to HS before birth, which should be verified in ongoing fully standardized experiments for HS and non-HS groups and consideration of climatic effects during early gestation. 

## Figures and Tables

**Table 1:** Metabolite specific models (model 1–3) selected according to the Akaike information criterion (AIC).

Source	Metabolite	Model	Abbreviation
Plasma Samples of calves	PC aa C34:1	y_ij_ = µ + c_i_ + e_ij_	(1)
	PC aa C38:3		
	PC aa C38:4		
	PC ae C38:3		
	PC ae C40:4		
	CE(20:3)		
	CE(20:4)		
	CE(22:5)		
	Hexoses		
	PC aa C38:1	y_ijk_ = µ + c_i_ + cc_j_ + e_ijk_	(2)
	p-Cresol-SO4		
	SM C18:0	y_ijk_ = µ + c_i_ + a_j_ + e_ijk_	(3)
	SM C18:1		
	SM C24:1		
	PC ae C30:2		

where y are the observations for blood metabolite concentrations (models (1)–(3)); µ is the overall mean effect (models (1)–(3)); c is the fixed effect of mTHI-class (no HS or HS) during the last week before birth (models (1)–(3)); cc is the fixed effect of calving condition (no birth assistance or minor birth assistance/dystocia) (model (2)); a is the fixed effect of age at blood sampling (blood sampling at age day 1; blood sampling at age days 2 or 3; blood sampling at age days 4 or 5; blood sampling at age days 6 or 7) (model (3)); and e is the random residual effect (models (1)–(3)).

**Table 2:** Individual mTHI classification, age at blood sampling (d), time of blood sampling, birthweight (kg) and calving condition of 47 female calves.

Calf	mTHI Class	Age (d)	Time of Blood Sampling	Birthweight (kg)	Calving Condition
2031	MHS_CALF_	3	11:40	43.0	no birth assistance
2037	MHS_CALF_	6	10:35	39.5	no birth assistance
2038	MHS_CALF_	1	10:37	42.5	minor birth assistance or dystocia
2039	MHS_CALF_	4	13:50	35.5	no birth assistance
2043	MHS_CALF_	5	11:22	39.0	no birth assistance
2044	MHS_CALF_	3	11:25	39.0	no birth assistance
2048	MHS_CALF_	4	10:30	37.0	no birth assistance
2052	MHS_CALF_	5	12:10	39.0	no birth assistance
2056	MHS_CALF_	7	11:10	39.0	no birth assistance
2059	MHS_CALF_	5	13:15	39.4	no birth assistance
2060	MHS_CALF_	3	13:10	37.0	minor birth assistance or dystocia
2064	MHS_CALF_	2	13:14	37.0	no birth assistance
2065	MHS_CALF_	2	13:15	38.5	no birth assistance
2068	MHS_CALF_	6	11:40	39.0	no birth assistance
2006	NMHS_CALF_	1	15:00	41.0	no birth assistance
2007	NMHS_CALF_	1	15:05	41.5	no birth assistance
2008	NMHS_CALF_	4	11:23	40.0	no birth assistance
2009	NMHS_CALF_	3	11:20	39.0	no birth assistance
2010	NMHS_CALF_	1	11:27	39.5	minor birth assistance or dystocia
2012	NMHS_CALF_	5	12:56	40.0	no birth assistance
2015	NMHS_CALF_	4	11:42	42.0	no birth assistance
2016	NMHS_CALF_	2	11:52	45.5	no birth assistance
2017	NMHS_CALF_	7	12:02	39.0	no birth assistance
2018	NMHS_CALF_	2	12:00	46.0	no birth assistance
2019	NMHS_CALF_	6	10:50	48.0	no birth assistance
2020	NMHS_CALF_	3	10:55	39.4	minor birth assistance or dystocia
2026	NMHS_CALF_	4	15:15	43.4	no birth assistance
2027	NMHS_CALF_	6	13:17	41.7	no birth assistance
2029	NMHS_CALF_	1	10:40	43.0	no birth assistance
2030	NMHS_CALF_	4	13:35	36.5	no birth assistance
2032	NMHS_CALF_	2	10:01	42.2	minor birth assistance or dystocia
2034	NMHS_CALF_	1	10:00	44.7	no birth assistance
2036	NMHS_CALF_	3	10:10	34.8	minor birth assistance or dystocia
2073	NMHS_CALF_	5	12:25	38.0	no birth assistance
2074	NMHS_CALF_	2	12:26	35.5	no birth assistance
2078	NMHS_CALF_	7	13:07	39.0	N/A
2080	NMHS_CALF_	6	13:06	35.0	no birth assistance
2085	NMHS_CALF_	4	12:20	NA	no birth assistance
2088	NMHS_CALF_	7	13:45	38.0	no birth assistance
2089	NMHS_CALF_	2	13:47	37.0	no birth assistance
2090	NMHS_CALF_	1	14:35	37.5	N/A
2102	NMHS_CALF_	1	11:32	38.5	no birth assistance
2104	NMHS_CALF_	6	14:20	42.5	no birth assistance
2107	NMHS_CALF_	7	11:45	42.0	N/A
2108	NMHS_CALF_	7	11:42	38.5	N/A
2109	NMHS_CALF_	6	11:39	39.5	N/A
2110	NMHS_CALF_	4	11:35	41.0	N/A

N/A = not available.

**Table 3:** Dam group data for parities, mean dry off weight of multiparous (mp) dams (kg), mean dry off milk yield of mp dams (kg) and mean weight of primiparous (pp) dams before birth (kg).

Dam Group	Parities	Mean Dry off Weight of mp Dams (kg)	Mean Dry off Milk Yield of mp Dams (kg)	Mean Weight of pp Dams before Birth (kg)
Dams of MHS_CALVES_	1–4	754.8 ± 18.6	25.1 ± 4.7	708.2 ± 49.9
Dams of NMHS_CALVES_	1–5	754.5 ± 87.3	23.3 ± 6.3	705.1 ± 62.7

**Table 4:** Phenotypic correlations between metabolite concentrations and birth weight in calves.

Metabolite	Birth Weight	*p*-Value
PC aa C34:1	0.15	0.3332
PC aa C38:3	0.14	0.3543
PC aa C38:4	0.22	0.1352
PC ae C38:3	0.20	0.1869
PC ae C40:4	0.26	0.0865
CE(20:3)	0.27	0.0669
CE(20:4)	0.26	0.0768
CE(22:5)	0.15	0.3233
Hexoses	0.24	0.1121
PC aa C38:1	0.26	0.0772
p-Cresol-SO4	−0.08	0.6019
SM C18:0	0.17	0.2481
SM C18:1	0.14	0.3643
SM C24:1	0.05	0.7256
PC ae C30:2	0.18	0.2288

## Data Availability

For scientific purposes, access will be provided upon written request to the corresponding author.

## Supplementary Materials

The following supporting information can be downloaded at: https://www.mdpi.com/article/10.3390/metabo13040494/s1, Table S1: Concentrations (µM) for all 259 metabolites. Values below the limit of detection (LOD) were replaced with < LOD. Table S2: Effects of fixed effects in models 1–3 on metabolite concentrations of calves. The table includes log transformed Least-squares means (LSMeans) with corresponding SE, number of observations (n) and *p*-value, adjusted with Bonferroni correction.
