# Supplementary material for: A Pilot Study on Across-Generation Impacts of Maternal Heat Stress on Blood Metabolites of Female Holstein Dairy Calves
Source: Metabolites. 2023 Mar 29;13(4):494. doi: 10.3390/metabo13040494 (PMC10141042; doi:10.3390/metabo13040494)
Supplement: Supplementary file 1 [file metabolites-13-00494-s001.zip › Table S1.pdf]

Supplementary Materials:

Table S1: Concentrations (µM) for all 259 metabolites. Values below the limit of detection (LOD) were replaced with < LOD.

Calf	2031	2037	2038	2039	2043	2044	2048	2052	2056	2059	2060	2064	2065	2068	2006	2007	2008	2009	2010	2012	2015	2016	2017	2018	2019	2020	2026	2027	2029	2030	2032	2034	2036	2073	2074	2078	2080	2085	2088	2089	2090	2102	2104	2107	2108	2109	2110
**mTHI class**	1	1	1	1	1	1	1	1	1	1	1	1	1	1	2	2	2	2	2	2	2	2	2	2	2	2	2	2	2	2	2	2	2	2	2	2	2	2	2	2	2	2	2	2	2	2	2
**Metabolilte**																																															
Carnitine	11.40	11.69	18.20	12.49	16.19	7.76	17.16	12.63	12.08	7.83	17.09	23.18	21.56	9.19	9.80	12.69	7.61	6.85	18.99	16.80	10.65	21.57	17.41	15.02	9.86	50.05	5.46	12.20	7.55	17.48	16.45	14.64	11.12	11.44	9.57	11.33	12.73	19.75	11.60	30.73	12.30	19.68	8.56	15.14	16.17	9.49	11.21
Acetylcarnitine	1.81	1.56	2.61	1.42	1.60	0.99	2.31	1.94	1.26	0.98	2.97	2.59	1.64	0.96	1.32	1.75	1.31	1.15	2.03	1.63	1.03	4.09	1.56	4.56	0.95	7.64	0.53	0.95	0.89	2.02	0.99	1.19	1.14	0.92	0.91	1.14	1.40	2.01	1.29	2.82	1.64	2.60	0.84	1.39	2.05	1.12	1.12
Propionylcarnitine	0.19	0.19	0.18	< LOD	0.19	< LOD	0.35	< LOD	< LOD	< LOD	0.27	0.50	0.34	< LOD	0.19	0.28	0.14	0.08	0.25	0.24	0.10	0.29	0.18	0.24	0.13	0.56	0.07	< LOD	< LOD	0.18	0.19	0.18	0.25	< LOD	< LOD	< LOD	0.19	0.33	< LOD	0.42	0.26	0.23	< LOD	< LOD	0.27	< LOD	0.23
Butyrilcarnitine	0.15	0.10	0.25	0.12	0.17	0.10	0.28	0.14	0.15	0.14	0.42	0.39	0.28	0.12	0.29	0.35	0.16	< LOD	0.28	0.15	< LOD	0.55	< LOD	0.24	0.14	0.34	< LOD	0.12	0.15	0.17	0.26	0.19	0.30	0.13	0.18	0.09	0.17	0.32	0.13	0.30	0.29	0.27	0.10	0.13	0.19	0.12	0.18
Trimethylamine N-oxide	8.90	6.50	2.56	55.92	39.72	1.97	1.47	4.85	26.80	5.99	4.72	3.77	4.57	23.54	3.03	6.11	5.61	3.79	1.34	5.36	12.90	2.92	5.39	2.28	8.45	2.83	2.59	10.39	2.75	8.24	6.12	1.67	3.46	5.69	3.93	5.31	5.93	6.61	15.15	18.17	1.16	10.10	2.72	15.52	7.25	5.67	4.01
1-Methylhistidine	1.64	2.75	10.97	4.80	5.83	1.82	2.17	2.43	5.47	2.41	3.00	3.41	2.24	3.82	6.15	4.91	3.56	3.80	6.84	3.66	4.20	20.75	5.80	9.40	2.74	3.36	3.42	5.46	4.70	3.47	2.18	17.68	2.72	2.64	2.42	2.81	2.79	2.90	5.77	3.93	4.20	15.61	2.91	3.54	2.81	3.78	3.15
3-Methylhistidine	1.20	2.02	7.25	2.47	3.18	1.36	1.11	1.72	3.04	1.75	2.36	1.58	1.09	2.39	2.60	2.69	1.65	1.71	4.16	2.13	2.20	10.14	3.71	7.09	1.30	1.45	2.16	3.52	2.69	2.25	1.11	5.12	1.37	1.36	1.42	1.54	1.84	1.79	3.40	2.34	3.11	8.05	1.62	1.75	1.82	2.58	2.45
5-Aminovaleric acid	0.44	0.29	0.47	0.84	0.35	0.14	0.25	0.38	0.15	0.35	0.45	0.54	0.54	0.25	0.73	0.34	0.36	0.24	0.67	0.65	0.18	0.15	0.54	1.55	0.29	0.44	0.11	0.47	0.32	0.20	2.00	0.46	1.21	0.96	0.62	2.29	2.24	0.24	1.33	2.40	0.37	0.41	0.47	0.25	1.12	0.13	0.10
α -Aminobutyric acid	2.44	2.85	2.79	9.80	10.68	4.08	6.85	3.90	6.73	2.79	5.76	7.80	7.20	6.24	6.14	6.13	3.80	6.25	14.77	8.30	7.46	5.82	4.70	3.78	9.34	5.22	4.24	11.07	3.80	4.89	4.44	2.59	7.05	3.08	6.17	4.03	4.85	14.04	8.50	8.04	10.30	7.07	6.31	4.38	5.29	6.34	6.88
Asymmetric dimethylarginine	0.93	1.49	0.65	1.57	2.58	0.91	1.84	1.26	2.08	1.48	1.75	1.73	1.35	1.89	1.84	1.53	1.36	1.12	1.15	2.21	1.45	0.92	1.72	1.23	1.42	1.95	1.25	2.11	0.62	1.40	1.05	0.45	1.71	1.48	1.46	1.31	1.79	2.29	1.90	2.29	1.70	1.20	2.04	1.85	2.14	2.16	1.78
α-Aminoadipic acid	1.15	2.34	0.65	1.32	2.06	2.01	4.28	1.41	2.64	2.98	2.56	3.17	3.21	1.81	4.35	4.08	1.77	1.38	1.84	2.65	1.08	4.09	2.47	0.70	2.88	2.02	1.06	1.75	0.90	2.45	1.29	0.86	3.76	3.11	3.89	2.03	2.61	3.12	1.67	2.97	3.02	2.13	3.67	1.62	2.99	2.51	3.92
Anserine	0.20	0.28	0.29	0.31	0.28	0.17	0.20	0.19	0.24	0.15	0.27	0.26	0.14	0.26	0.24	0.14	0.24	0.19	0.21	0.26	0.23	0.33	0.25	0.31	0.23	0.25	0.27	0.23	0.13	0.33	0.17	0.21	0.21	0.27	0.18	0.12	0.20	0.13	0.53	0.22	0.27	0.35	0.26	0.25	0.38	0.31	0.34
β-Aminobutyric acid	0.05	0.06	0.40	0.08	0.06	0.08	0.03	0.04	0.05	0.03	0.11	0.31	0.16	0.04	0.22	0.13	0.14	0.13	0.82	0.13	0.07	0.50	0.24	0.34	< LOD	0.14	0.07	0.06	0.28	0.15	0.37	0.45	0.07	0.05	0.15	0.04	0.03	0.06	0.07	0.11	0.24	0.85	< LOD	0.05	0.06	0.04	0.07
Betaine	66.98	140.87	182.97	95.57	200.42	83.57	98.78	87.28	175.03	105.10	125.28	120.80	129.70	121.77	96.77	105.27	69.61	68.71	50.69	97.33	93.02	171.27	140.00	60.81	65.68	97.58	49.86	257.84	107.98	152.80	122.54	162.11	140.50	143.02	153.61	175.18	139.25	159.27	136.83	296.08	106.21	231.37	133.82	176.60	144.53	176.83	107.17
Carnosine	4.91	5.78	6.21	8.12	9.61	4.86	6.18	5.62	7.98	5.83	7.97	7.24	5.58	12.84	8.74	6.81	7.41	8.52	5.90	8.57	8.16	16.00	8.14	10.66	11.63	8.74	8.73	9.43	3.30	12.53	4.74	4.81	12.43	10.51	5.33	7.91	5.36	9.00	22.34	12.43	9.02	9.03	9.54	10.41	12.84	14.05	10.71
Citrulline	40.52	71.27	36.13	19.99	39.09	25.48	65.34	34.37	48.98	64.24	37.56	104.32	118.60	45.90	148.24	107.01	63.71	57.43	42.21	70.78	34.36	79.81	70.88	61.45	86.98	84.03	42.21	49.96	48.21	75.55	84.88	31.52	159.23	49.42	110.91	91.47	95.10	47.77	44.99	161.72	74.12	103.66	83.04	45.72	105.74	86.87	82.52
Creatinine	49.72	62.91	103.47	64.33	79.48	43.39	51.68	49.17	88.31	67.82	88.97	62.80	53.97	75.77	95.65	78.30	101.92	66.45	79.28	83.11	63.80	183.27	73.80	80.95	67.13	79.28	66.94	74.90	72.39	74.25	53.42	98.45	64.00	62.69	63.78	46.45	60.51	52.12	80.28	75.86	69.48	115.81	64.87	70.07	85.58	80.38	72.53
Cystine	11.61	30.15	12.84	19.75	31.35	13.95	28.41	19.41	37.03	24.58	18.72	17.26	18.88	20.18	26.58	26.30	16.37	15.87	11.61	27.86	18.72	18.71	28.74	24.91	24.23	17.51	13.00	35.76	11.66	19.74	19.30	10.18	20.78	27.62	20.90	27.59	34.88	37.04	32.56	30.94	30.84	20.91	30.91	34.54	30.65	30.51	27.64
Homoarginine	2.82	4.88	3.16	2.05	5.74	2.45	2.65	2.53	1.64	3.52	4.69	3.94	2.45	2.27	6.34	9.01	1.51	1.97	2.38	6.43	2.46	7.19	9.07	6.43	4.03	4.28	1.47	1.23	1.91	2.87	1.37	0.86	3.34	4.41	5.13	5.45	5.07	2.69	3.16	4.51	3.98	4.29	5.11	2.38	6.72	3.20	3.61
Homocysteine	3.20	8.44	6.98	4.94	8.81	4.77	5.64	4.29	7.59	7.08	8.33	6.05	5.31	6.68	8.37	6.69	6.50	7.97	9.42	14.52	5.37	8.00	10.51	6.54	12.08	10.97	5.58	6.99	3.92	5.94	5.75	3.55	9.77	17.84	4.80	9.89	11.51	8.76	8.33	9.69	9.06	12.64	8.80	8.60	13.49	10.89	7.05
Kynurenine	2.74	7.71	1.16	5.63	7.84	1.98	3.21	1.82	5.72	2.88	2.37	2.44	2.10	5.39	3.65	2.90	4.18	1.90	1.47	4.63	3.65	2.94	5.81	0.74	2.47	3.24	2.53	7.79	2.05	2.73	2.82	1.58	3.18	3.61	2.65	1.78	2.43	4.40	10.17	2.54	1.71	2.49	2.71	5.29	2.42	2.92	3.08
Methionine sulfoxide	0.91	2.63	0.77	0.64	2.40	0.87	1.08	0.70	1.59	1.50	2.78	2.24	4.60	1.55	5.24	2.52	1.26	0.57	1.61	3.62	0.39	1.95	3.77	0.59	1.51	3.99	0.64	2.36	0.48	1.26	0.85	0.37	1.75	1.93	1.40	2.77	3.08	2.78	1.67	3.46	2.71	1.88	1.71	1.49	3.15	1.96	1.30
Ornithine	49.05	88.22	27.93	46.33	120.36	53.48	94.35	62.34	85.16	107.07	73.80	163.50	189.62	102.76	188.95	150.26	78.01	67.12	73.51	100.78	41.93	40.76	105.27	97.18	90.88	106.17	65.59	46.33	42.24	64.04	82.77	17.49	241.85	79.71	140.79	152.15	137.39	94.47	76.10	152.21	159.13	87.47	115.64	94.29	125.52	132.44	142.32
Phenylacetylglycine	0.23	0.32	0.73	0.17	0.58	0.05	0.08	0.24	0.21	0.36	0.19	0.13	0.31	0.16	0.34	0.34	0.44	0.17	0.46	0.32	0.07	0.16	0.77	0.81	0.35	0.32	0.09	0.21	0.56	0.22	0.91	0.36	0.34	0.51	0.15	0.47	0.22	0.25	1.00	1.21	0.35	0.35	0.12	0.37	0.62	0.49	0.09
Pro Betaine	0.10	0.34	0.14	0.11	0.11	0.09	0.07	0.13	0.20	0.16	0.18	0.15	0.13	0.11	0.13	0.13	0.17	0.11	0.17	0.25	0.11	0.37	0.25	0.14	0.12	0.27	0.07	0.20	0.13	0.18	0.12	0.14	0.09	0.22	0.11	0.26	0.21	0.12	0.30	0.21	0.11	0.41	0.12	0.18	0.36	0.16	0.15
Sarcosine	1.33	3.59	2.23	2.89	6.05	1.98	2.56	4.70	12.52	3.91	2.50	4.49	3.03	5.89	6.33	5.21	2.33	2.77	3.47	7.46	4.44	3.04	10.07	2.09	4.02	2.93	1.92	8.34	1.70	4.44	2.57	1.24	4.66	3.80	4.88	6.67	6.26	4.96	7.35	5.55	3.85	4.55	4.83	12.14	7.66	6.42	4.96
Symmetric dimethylarginine	0.66	1.05	0.86	0.91	1.74	0.52	0.68	0.78	1.24	0.66	1.11	0.78	0.55	0.95	1.42	1.36	0.81	0.61	0.95	1.09	0.80	1.60	1.34	1.33	0.60	0.99	0.91	1.63	0.48	0.85	0.53	0.81	0.67	0.72	0.68	0.56	0.73	0.86	1.27	1.15	0.88	1.65	0.82	1.11	1.14	0.93	0.86
trans-4-Hydroxyproline	22.83	62.75	27.16	79.95	131.99	31.27	52.69	42.30	82.55	44.36	48.58	41.76	35.70	109.27	46.35	37.99	31.03	52.18	21.06	61.40	80.20	32.72	53.78	58.01	55.57	44.10	52.18	45.44	16.01	70.33	18.93	17.64	40.79	59.51	32.03	58.21	55.50	59.29	84.99	54.42	41.89	46.68	68.69	67.77	62.98	51.06	52.48
Taurine	30.12	64.11	55.87	50.66	143.85	43.57	82.35	56.60	81.41	40.44	64.11	49.51	37.73	58.58	88.58	79.02	49.37	42.10	49.96	48.37	92.36	167.20	65.39	84.50	44.09	77.23	25.18	73.70	44.09	79.64	51.08	47.22	80.58	31.38	47.95	34.19	36.69	59.52	98.71	104.70	56.60	118.67	30.35	70.25	52.30	47.47	64.58
Alanine	144.02	220.20	133.58	428.92	661.65	217.07	335.00	171.15	400.75	188.89	289.08	339.17	282.82	362.13	315.67	343.85	218.90	323.18	322.25	380.49	533.63	335.40	293.12	217.96	319.43	370.16	278.09	373.61	109.58	306.82	149.24	104.05	295.34	183.68	240.03	232.73	286.99	506.15	344.59	383.45	390.61	532.74	324.14	279.15	259.72	281.19	294.49
Arginine	144.42	236.13	57.03	86.76	213.99	99.19	225.59	135.98	134.93	199.23	79.06	530.23	328.89	146.53	332.87	292.11	128.10	147.51	114.52	246.50	144.60	124.22	328.99	204.77	265.91	274.65	123.25	65.99	74.95	140.20	195.02	43.43	290.94	223.48	285.67	377.38	331.00	181.31	145.95	513.77	221.88	201.17	277.10	255.41	351.06	221.88	290.91
Asparagine	33.15	50.49	28.53	39.61	75.22	36.33	71.12	46.39	45.36	46.39	51.83	109.81	99.65	63.93	149.03	130.76	47.69	56.73	64.32	60.09	41.15	66.25	75.67	50.19	50.38	62.50	29.90	48.03	29.76	54.08	41.56	22.17	158.04	46.59	112.89	65.06	78.51	94.41	54.97	118.15	105.13	112.14	60.28	46.66	57.97	70.49	84.11
Aspartate	4.57	4.38	4.32	7.03	14.63	5.91	16.63	4.37	11.62	7.89	10.62	13.23	11.62	10.42	10.77	9.43	6.17	6.97	3.29	9.72	8.66	9.18	8.94	7.53	7.14	8.72	4.52	8.58	5.05	10.22	4.76	4.73	9.96	5.83	9.39	4.35	6.97	9.61	10.74	12.69	9.54	12.23	7.05	7.88	6.50	6.31	11.21
Cysteine	17.62	46.84	23.86	32.21	51.81	21.94	37.74	24.74	51.77	38.60	31.61	30.31	28.95	33.10	42.35	39.89	32.47	36.16	31.61	46.18	34.62	35.97	46.84	32.66	47.81	42.90	29.33	43.41	18.34	33.47	27.61	15.33	33.14	47.79	29.64	39.59	50.98	56.37	49.10	47.06	39.22	37.93	48.96	52.47	54.20	48.02	48.76
Glutamine	112.68	190.43	238.88	279.45	429.31	172.40	274.94	244.52	397.76	215.22	267.05	554.39	451.85	527.34	818.82	639.13	245.46	297.33	478.88	339.02	286.22	460.36	331.61	262.14	326.05	276.96	83.92	325.65	212.97	366.21	260.29	171.27	631.01	276.07	564.53	370.72	352.69	833.84	306.68	517.85	618.66	859.54	306.68	303.49	226.03	387.33	437.20
Glutamate	50.58	78.55	50.69	93.95	149.67	61.07	186.81	60.41	158.41	83.25	127.82	99.20	123.45	142.02	183.10	90.67	96.74	95.47	194.85	122.39	119.46	152.75	162.54	105.75	119.46	139.04	60.32	134.37	66.09	133.28	56.81	71.78	109.25	94.72	50.25	98.10	145.30	105.86	118.09	122.06	167.71	115.11	122.06	136.94	134.96	120.07	141.91
Glycine	111.24	242.61	131.37	464.04	820.01	166.33	331.61	278.63	691.82	280.75	220.36	244.73	216.13	556.21	317.98	353.42	136.96	252.85	190.60	306.49	714.50	283.50	359.17	297.87	244.23	261.47	299.78	509.59	125.01	268.04	139.85	103.72	194.94	250.03	248.97	329.49	328.43	431.19	563.89	295.03	349.41	344.37	444.06	417.88	295.03	348.40	338.33
Histidine	54.34	60.58	95.70	45.29	172.08	83.43	93.99	121.77	67.53	99.12	152.96	207.30	152.96	103.65	282.84	298.12	92.11	111.80	210.22	83.04	125.17	217.86	89.15	212.13	56.28	79.21	46.44	74.77	75.37	77.09	140.89	84.13	302.91	107.68	251.58	98.02	131.83	165.04	71.99	285.34	280.30	223.84	100.02	72.19	86.41	132.08	179.47
Isoleucine	82.70	132.49	56.82	96.78	153.19	80.11	142.84	89.22	119.03	129.38	148.01	212.18	159.40	124.21	187.84	192.58	113.84	99.61	85.00	111.00	54.64	77.03	127.12	93.73	111.00	142.30	108.15	108.68	50.61	113.85	93.57	32.40	212.18	110.75	154.22	119.03	142.84	157.33	83.27	206.12	141.52	124.08	140.49	110.75	145.62	159.98	174.33
Leucine	99.77	176.72	103.29	88.91	172.33	117.45	187.69	120.74	145.98	174.52	198.67	320.50	238.18	167.94	315.60	335.20	144.39	121.70	174.30	125.83	59.41	145.42	174.30	139.24	121.70	172.24	126.86	133.91	104.82	126.23	117.45	53.34	319.41	138.30	243.67	174.52	199.77	255.74	135.06	286.07	236.09	223.33	156.33	138.25	170.16	214.82	238.22
Lysine	95.05	228.66	46.25	62.45	155.86	96.18	147.66	129.20	122.02	145.60	162.01	325.05	158.93	132.27	236.26	307.58	97.18	54.12	93.61	182.76	58.75	103.42	254.09	94.50	133.73	147.10	80.77	133.30	51.17	124.07	78.75	25.74	195.85	186.62	234.81	244.04	247.12	210.20	176.06	344.26	219.34	135.74	178.03	144.59	299.99	178.03	224.26
Methionine	30.44	171.34	21.76	35.17	69.78	35.28	49.15	34.94	47.23	50.73	48.36	75.30	65.27	55.35	107.18	88.87	25.18	36.40	33.97	161.77	29.73	55.81	183.01	47.52	91.30	51.26	42.57	116.11	17.59	32.92	29.42	8.59	72.59	89.39	55.12	156.69	139.78	69.33	71.98	79.63	69.47	77.01	80.94	49.92	152.92	61.39	59.75
Phenylalanine	38.07	103.42	38.07	49.18	102.98	33.12	43.46	61.06	58.20	63.48	85.27	84.94	58.42	96.71	76.16	85.81	61.92	37.03	52.36	61.52	72.57	66.60	83.22	56.94	32.35	67.49	74.96	68.76	29.27	39.06	40.71	22.01	63.15	64.03	54.24	84.17	86.04	75.70	90.93	90.21	55.68	55.99	62.12	68.65	76.42	67.33	103.19
Proline	99.76	136.82	102.02	165.91	233.78	135.74	160.52	129.28	144.36	133.59	221.93	350.13	199.30	192.84	498.71	428.89	124.68	158.59	238.38	162.58	164.57	279.28	160.58	167.57	144.63	171.56	140.64	147.59	140.05	161.60	145.44	99.76	360.90	115.27	263.94	173.45	165.91	225.16	165.53	284.50	344.50	392.09	210.01	123.11	157.25	174.84	235.88
Serine	57.12	116.36	45.38	110.02	186.18	68.87	131.17	77.86	142.81	118.48	99.54	156.56	129.06	162.91	170.58	193.32	74.68	78.66	77.23	115.61	128.88	130.78	142.15	71.07	134.57	137.41	103.29	151.27	58.71	88.54	59.35	42.53	170.31	88.54	148.10	137.52	161.85	138.58	132.59	140.75	145.85	148.90	138.71	101.28	142.79	132.59	140.75
Threonine	49.92	117.39	64.41	55.74	89.68	43.04	88.63	55.63	116.34	83.97	84.18	172.39	142.78	107.88	195.36	215.87	47.47	79.02	91.82	70.82	83.52	171.92	139.68	83.81	99.63	96.90	52.75	73.08	75.30	61.55	130.08	66.63	161.81	92.86	156.52	162.87	122.68	129.03	95.59	150.60	172.56	202.89	234.26	157.92	121.31	115.04	100.61
Tryptophan	28.23	59.19	34.36	25.49	40.05	24.62	34.36	32.17	35.34	50.22	37.75	51.64	67.40	36.87	68.56	59.19	31.01	31.79	49.54	43.01	29.06	91.86	52.47	33.25	39.89	49.35	40.57	43.33	27.90	35.89	32.61	21.45	52.08	31.18	50.11	40.92	37.64	51.75	40.64	65.04	48.29	81.80	46.43	56.56	47.98	45.50	52.84
Tyrosine	55.07	130.77	46.89	38.06	70.06	49.44	25.73	40.51	39.66	54.01	111.63	123.33	73.46	52.84	107.94	121.44	66.50	53.97	66.21	61.01	35.76	182.15	69.58	45.97	32.09	82.69	88.86	44.01	38.70	44.65	51.56	18.07	124.39	56.13	62.09	88.14	64.11	57.84	58.16	87.80	106.77	111.96	79.10	53.59	82.41	55.88	99.83
Valine	125.94	192.38	139.82	137.84	224.11	159.65	282.62	174.53	194.36	247.91	278.65	461.11	368.89	245.93	439.70	438.76	213.25	211.36	283.07	195.32	134.93	289.68	213.25	211.36	231.18	246.27	183.05	155.69	204.28	189.40	265.76	147.75	474.00	195.35	388.72	224.11	255.84	329.22	174.23	331.92	421.47	329.00	249.18	160.61	212.19	273.52	328.03
Glycocholic acid	1.75	5.84	1.13	0.78	3.22	2.93	2.71	1.32	3.79	1.18	8.64	2.25	5.19	3.69	3.77	4.38	1.83	1.85	1.34	1.44	0.60	0.73	1.28	5.22	1.53	0.95	1.78	5.77	0.88	0.63	1.05	0.39	1.55	3.66	3.91	1.73	3.81	7.18	5.87	2.90	1.82	1.05	3.30	4.57	5.78	1.90	1.13
Glycochenodeoxycholic acid	0.76	2.67	2.54	0.35	3.06	1.55	1.96	3.09	2.03	1.43	5.56	2.16	2.45	1.95	2.37	2.86	2.71	0.94	1.18	2.20	0.62	0.66	1.55	6.93	0.91	1.93	1.55	2.76	1.16	0.73	0.74	1.11	1.08	2.96	1.26	2.11	2.99	2.91	4.41	1.45	1.80	0.74	3.07	2.07	2.48	1.98	1.64
Glycoursodeoxycholic acid	0.01	0.05	0.01	< LOD	0.03	0.02	0.02	0.09	0.02	0.04	0.01	0.01	0.13	0.02	0.03	0.02	0.03	0.02	0.06	0.01	< LOD	< LOD	0.08	0.12	< LOD	0.03	0.03	0.02	0.00	0.01	0.08	< LOD	0.07	0.04	0.03	0.01	0.01	0.04	0.01	0.03	0.04	< LOD	0.03	< LOD	< LOD	< LOD	0.02
Taurocholic acid	2.67	7.47	0.93	1.02	3.26	3.02	2.45	2.34	2.94	2.07	7.51	2.67	4.47	4.42	2.95	4.34	4.14	4.49	2.40	3.19	0.78	0.84	1.25	8.94	2.08	1.57	2.27	1.56	0.45	2.49	1.43	0.36	3.00	5.27	3.05	2.22	5.36	7.78	8.35	3.69	2.71	1.25	3.74	7.64	7.30	2.70	1.64
Taurochenodeoxycholic acid	1.06	3.70	3.08	0.67	4.61	2.09	2.30	1.53	2.85	2.22	8.11	2.14	2.39	3.75	3.14	4.85	7.02	2.58	3.62	6.22	1.31	1.39	1.86	15.78	2.24	3.32	1.86	1.47	1.44	2.95	1.26	1.72	1.70	2.62	1.19	2.25	5.76	4.39	10.29	1.88	2.93	0.87	3.85	4.44	5.56	2.26	2.72
β-Alanine	1.63	5.96	1.25	6.28	4.16	1.60	2.04	2.30	5.20	2.07	1.87	2.31	1.03	4.44	0.87	1.04	0.90	1.50	0.80	3.51	2.71	1.45	6.48	1.62	6.00	3.60	6.04	7.75	0.59	12.89	1.22	0.86	2.07	4.34	0.92	5.36	1.93	2.49	12.89	3.27	1.26	1.10	3.63	8.08	7.29	4.44	< LOD
γ-Aminobutyric acid	0.10	0.16	0.14	0.18	0.28	0.10	0.15	0.13	0.21	0.15	0.20	0.17	0.20	0.15	0.13	0.14	0.11	0.14	0.14	0.19	0.16	0.15	0.24	0.12	0.13	0.18	0.10	0.26	0.10	0.18	0.16	0.16	0.20	0.19	0.14	0.13	0.13	0.24	0.25	0.23	0.14	0.16	0.19	0.21	0.21	0.13	0.16
Putrescine	< LOD	0.13	0.11	0.17	0.23	0.11	0.19	0.09	0.13	0.22	0.15	0.12	0.14	0.16	0.13	0.09	< LOD	0.11	0.13	0.12	0.13	0.11	< LOD	< LOD	0.09	< LOD	0.08	0.19	0.14	0.13	0.14	0.11	0.14	0.11	0.15	< LOD	0.11	0.13	0.17	0.16	0.11	0.12	0.10	0.14	0.12	0.10	0.13
Serotonin	0.07	0.13	0.23	0.33	0.10	0.09	0.07	0.14	1.05	0.36	0.46	1.14	0.54	0.31	0.06	< LOD	< LOD	0.08	0.09	0.06	0.07	0.10	0.06	0.14	0.14	0.13	0.06	0.06	0.08	0.21	0.09	0.13	0.22	0.33	2.28	0.10	0.39	0.20	0.30	0.22	0.10	0.28	0.29	0.43	0.33	0.18	0.94
Hexoses (including glucose)	3976.95	7104.68	4573.96	6005.30	7217.54	5680.80	8354.85	4128.80	5167.20	8786.16	7783.48	6827.96	8496.13	6243.25	8179.00	7092.92	8012.79	9292.16	15771.97	11596.29	8441.56	9754.92	6359.27	10097.82	10427.57	8508.72	8962.02	7991.40	5893.34	7522.19	5686.35	7088.98	6783.40	6775.14	5834.14	5809.35	6828.70	6254.54	7363.35	7387.30	7304.25	9065.24	10961.55	7432.09	6758.24	6817.16	7620.96
Aconitic acid	6.53	33.27	20.20	16.94	21.96	16.90	21.26	8.09	18.93	23.49	32.60	22.73	16.75	15.46	11.59	9.98	19.45	17.58	10.09	15.60	15.81	29.47	11.08	25.67	15.77	23.03	12.85	10.05	4.71	29.49	6.49	14.28	13.46	13.72	10.86	9.77	16.00	13.81	20.12	25.83	11.04	22.99	19.09	15.67	19.41	12.51	9.50
Tetradecanedioic acid	0.15	0.27	< LOD	0.22	0.11	0.16	0.14	< LOD	< LOD	0.10	1.58	0.13	0.13	0.16	0.19	0.20	0.53	0.48	0.12	< LOD	0.13	0.30	0.12	0.35	0.14	0.16	0.24	< LOD	0.22	0.13	< LOD	< LOD	0.17	0.11	0.12	0.14	0.18	0.11	0.25	0.19	0.32	0.31	0.17	0.10	0.25	0.15	0.11
Hippuric acid	2.75	4.10	3.38	1.06	2.41	1.92	4.25	3.68	2.06	5.20	3.71	1.95	3.74	2.20	3.36	2.32	3.84	3.04	4.61	4.44	1.15	5.92	5.51	5.70	3.70	7.66	1.23	3.38	3.64	2.68	2.81	3.34	1.98	2.11	2.84	1.22	1.83	2.91	2.50	10.46	1.73	3.79	0.77	1.46	3.45	3.17	1.68
Lactic acid	1600.60	4339.12	4772.94	5383.70	4619.62	2686.81	4158.87	1288.76	2195.89	3880.78	4909.37	6076.97	4479.44	1863.18	4197.51	5343.37	2347.99	3026.83	5979.48	3492.78	3885.26	4415.22	3959.33	2455.44	2536.67	3023.46	5407.09	2580.87	2772.71	4518.43	3952.00	2180.15	4316.74	2061.67	2686.01	2206.14	5121.44	2601.54	4188.73	3577.75	2606.11	4123.69	2571.77	1859.61	2699.86	1978.70	3395.72
Cer(d16:1/23:0)	0.08	0.13	0.02	0.04	0.04	0.13	0.11	0.04	< LOD	0.10	0.21	0.25	0.06	0.04	0.04	0.04	0.07	0.08	< LOD	0.09	0.05	0.06	0.05	0.08	< LOD	0.09	0.06	< LOD	< LOD	0.05	0.04	0.06	0.19	0.20	0.06	0.16	0.09	0.03	0.03	0.12	0.08	0.11	0.06	0.08	0.16	0.12	0.16
Cer(d16:1/24:0)	0.04	0.06	0.02	0.02	0.02	0.07	0.02	0.04	< LOD	0.03	0.14	0.08	0.06	< LOD	0.06	0.05	0.10	0.09	0.05	0.09	0.04	0.09	0.04	0.11	0.05	0.08	0.04	< LOD	0.02	0.02	0.03	0.04	0.09	0.07	0.04	0.08	0.05	0.03	0.03	0.06	0.03	0.04	0.08	< LOD	0.06	0.06	0.06
Cer(d18:1/16:0)	0.12	0.21	0.12	0.16	0.12	0.16	0.14	0.15	0.14	0.23	0.35	0.25	0.14	0.13	0.13	0.23	0.32	0.25	0.17	0.26	0.21	0.21	0.17	0.24	0.22	0.22	0.24	0.15	0.13	0.21	0.11	0.09	0.29	0.19	0.19	0.27	0.26	0.13	0.15	0.22	0.14	0.21	0.27	0.24	0.36	0.27	0.32
Cer(d18:1/18:0)	0.03	0.06	0.04	0.04	0.04	0.04	0.03	0.03	0.05	0.05	0.12	0.07	0.03	0.02	0.05	0.07	0.09	0.06	0.06	0.08	0.07	0.09	0.05	0.10	0.05	0.05	0.06	0.05	0.05	0.05	0.03	0.03	0.04	0.03	0.04	0.04	0.05	0.04	0.04	0.05	0.03	0.07	0.03	0.06	0.05	0.05	0.05
Cer(d18:1/22:0)	0.10	0.16	0.07	0.06	0.07	0.16	0.07	0.09	0.07	0.15	0.28	0.26	0.10	0.08	0.10	0.14	0.23	0.27	0.07	0.21	0.14	0.14	0.11	0.23	0.09	0.14	0.16	0.11	0.06	0.09	0.07	0.07	0.22	0.16	0.11	0.24	0.24	0.10	0.10	0.16	0.10	0.08	0.21	0.22	0.32	0.22	0.22
Cer(d18:1/23:0)	0.10	0.22	0.06	0.07	0.06	0.11	0.09	0.08	0.08	0.13	0.30	0.24	0.09	0.11	0.10	0.18	0.31	0.21	0.09	0.24	0.19	0.14	0.15	0.29	0.13	0.14	0.24	0.07	0.04	0.16	0.06	0.05	0.27	0.18	0.11	0.35	0.29	0.11	0.10	0.16	0.05	0.05	0.19	0.17	0.45	0.27	0.30
Cer(d18:1/24:0)	0.14	0.34	0.14	0.07	0.13	0.19	0.19	0.17	0.15	0.23	0.46	0.38	0.20	0.26	0.19	0.21	0.42	0.41	0.15	0.32	0.24	0.19	0.20	0.48	0.25	0.23	0.24	0.18	0.12	0.22	0.09	0.18	0.31	0.35	0.19	0.49	0.55	0.17	0.20	0.18	0.12	0.17	0.35	0.32	0.59	0.34	0.37
Cer(d18:1/24:1)	0.10	0.26	0.11	0.15	0.09	0.12	0.19	0.10	0.11	0.12	0.37	0.27	0.11	0.15	0.16	0.22	0.26	0.21	0.16	0.15	0.22	0.23	0.14	0.38	0.20	0.26	0.26	0.11	0.14	0.13	0.09	0.06	0.29	0.13	0.23	0.18	0.23	0.10	0.11	0.23	0.10	0.12	0.16	0.18	0.20	0.20	0.24
CE(14:0)	19.35	15.54	5.04	15.16	7.03	7.46	21.14	13.45	7.21	23.47	35.61	26.34	16.28	11.65	15.14	12.98	25.04	24.27	11.45	26.23	15.61	9.43	14.82	21.20	31.30	24.74	25.81	11.20	7.03	16.68	13.00	4.73	18.78	10.99	12.79	20.10	18.25	16.24	15.98	43.94	11.76	11.84	19.89	15.12	20.46	28.17	28.03
CE(15:0)	5.67	4.98	2.31	5.16	2.75	2.57	6.06	5.35	2.51	7.92	8.34	6.74	5.21	5.01	4.93	5.00	7.18	7.55	6.30	12.69	6.06	5.00	4.29	6.48	12.41	8.16	7.82	3.95	3.26	5.99	2.99	2.28	5.12	4.47	4.17	5.72	6.41	6.50	4.84	9.51	4.13	5.00	5.06	4.29	4.77	8.92	11.75
CE(16:0)	31.55	36.79	22.34	38.02	25.83	22.19	40.52	34.63	27.62	46.56	66.44	52.93	36.27	34.27	33.26	44.85	41.52	46.82	51.43	61.30	41.30	40.80	39.11	51.51	70.67	51.35	52.38	46.86	33.14	31.82	25.59	25.78	43.55	29.81	37.04	47.14	48.27	50.43	38.37	70.57	28.90	38.00	47.80	45.94	54.81	53.58	66.11
CE(16:1)	16.95	14.34	11.15	16.30	16.33	10.95	29.50	23.78	9.88	40.88	58.59	40.16	26.24	15.22	40.90	38.43	45.62	43.90	45.26	35.48	29.89	27.65	16.44	47.98	51.33	34.56	43.29	21.46	18.73	19.88	17.91	13.70	35.61	13.22	32.79	25.07	22.01	27.54	14.07	64.96	25.56	27.57	23.41	24.32	15.46	36.84	51.03
CE(17:0)	1.75	2.00	1.53	1.97	1.45	1.04	2.82	2.41	0.96	3.09	2.29	2.52	1.97	2.64	1.64	2.49	2.64	3.47	3.74	5.64	1.77	2.31	2.12	4.24	5.58	3.07	2.72	3.21	1.54	2.37	1.01	1.48	2.88	1.99	1.63	1.61	3.18	2.87	1.94	3.12	1.37	1.86	1.95	2.33	2.41	3.46	4.70
CE(17:1)	2.54	2.08	1.84	3.14	1.94	1.63	3.09	3.09	1.32	4.89	4.70	4.85	3.67	3.09	4.18	4.96	5.06	6.01	5.81	6.83	4.50	3.77	2.72	5.91	8.32	4.94	5.93	3.18	2.41	3.61	2.58	2.07	3.70	2.57	3.84	3.25	3.35	4.43	1.93	5.09	3.04	3.50	2.75	3.08	2.44	4.80	7.00
CE(18:0)	1.39	1.69	1.35	1.55	1.46	1.19	2.64	2.47	1.05	2.85	2.09	2.30	1.70	1.68	1.59	2.18	2.08	2.63	2.78	3.62	1.68	2.06	1.91	3.32	3.41	2.60	2.15	2.29	1.57	1.89	1.13	1.22	2.34	1.69	1.70	2.53	2.46	2.50	1.73	3.33	1.76	1.77	2.10	2.37	2.68	2.66	4.50
CE(18:1)	25.17	37.23	21.97	33.95	32.84	17.61	42.51	44.75	26.55	57.19	55.49	52.11	46.80	46.56	36.37	51.76	50.80	53.13	67.62	80.90	39.45	37.56	47.54	74.64	95.08	70.22	57.08	52.27	27.94	37.96	25.71	26.21	44.47	37.97	39.45	62.53	52.21	52.53	34.41	76.64	34.30	42.56	52.58	58.96	56.94	58.61	93.37
CE(18:2)	88.25	223.90	17.18	105.16	108.31	55.35	131.86	129.31	206.94	278.03	203.49	168.45	97.64	230.53	73.60	63.97	165.03	190.64	51.83	430.43	165.74	28.33	338.19	99.43	372.67	158.39	245.04	270.11	32.59	182.39	47.06	21.81	101.28	255.51	83.07	568.41	351.41	230.20	352.84	243.16	74.00	51.85	471.97	422.66	537.30	491.32	379.84
CE(18:3)	32.12	30.17	6.84	35.97	22.43	14.93	37.39	40.02	17.34	74.33	52.40	49.56	26.78	43.80	15.98	10.43	37.46	44.89	18.96	167.20	54.61	8.59	31.15	42.93	100.58	60.22	73.31	29.47	7.23	53.27	20.68	5.35	31.07	36.66	23.70	46.94	59.33	55.72	31.23	80.52	24.57	15.84	46.72	46.61	38.11	114.55	110.95
CE(20:3)	1.63	1.54	1.19	1.28	2.14	1.42	1.84	1.63	1.54	3.40	4.34	3.98	3.07	2.01	4.25	4.63	3.74	3.44	4.58	4.41	3.59	3.99	2.29	2.90	6.23	3.08	4.46	3.10	3.75	1.82	1.92	2.52	2.81	2.27	4.04	3.29	2.55	3.84	2.19	4.68	3.05	4.07	3.80	2.46	2.84	3.93	4.77
CE(20:4)	9.38	10.55	6.51	9.43	12.79	7.75	14.27	9.00	8.80	21.36	27.81	20.34	16.02	16.28	19.20	22.87	19.97	23.98	20.79	29.57	18.59	24.58	14.41	12.55	41.00	18.05	28.07	25.64	18.84	12.10	8.54	13.72	17.65	11.15	15.20	24.70	17.90	25.90	18.75	28.09	10.59	17.80	27.23	20.74	22.21	30.90	24.94
CE(20:5)	19.99	19.75	12.76	22.30	14.00	9.89	17.45	18.87	12.85	29.64	26.73	35.88	22.56	32.53	8.01	7.79	12.13	15.84	41.33	80.08	20.94	9.50	23.19	28.22	49.86	38.07	21.72	16.77	7.87	24.84	21.55	8.16	19.39	20.03	10.47	23.52	28.48	41.17	23.02	46.25	14.96	21.99	17.94	17.99	26.66	31.22	34.42
CE(22:5)	0.27	0.42	0.38	0.25	0.33	< LOD	0.32	0.34	0.23	0.44	0.52	0.80	0.43	0.45	0.41	0.69	0.44	0.46	1.27	1.05	0.37	0.60	0.47	0.71	0.78	0.74	0.48	0.48	0.42	0.36	0.32	0.33	0.39	0.41	0.45	0.45	0.48	0.74	0.36	0.64	0.33	0.61	0.37	0.48	0.50	0.48	0.56
CE(22:6)	1.81	2.00	2.37	2.22	1.41	0.74	1.70	2.03	1.53	2.39	3.72	5.98	3.92	2.47	2.96	4.69	1.86	2.17	8.46	3.85	1.74	4.29	2.73	3.71	2.78	4.12	2.37	2.42	4.01	1.94	3.35	2.88	2.67	1.93	2.72	2.17	3.13	5.01	2.33	6.38	2.29	6.12	2.09	1.90	3.25	2.00	4.55

p-Cresol sulfate	0.72	1.39	0.69	0.37	1.97	0.20	0.17	0.32	0.91	2.41	0.27	0.14	0.17	0.65	3.80	1.78	1.85	1.19	0.60	1.52	0.09	1.58	6.12	0.38	1.18	0.93	0.07	0.83	1.59	0.75	1.09	1.84	0.41	5.30	0.45	3.11	0.90	0.74	5.26	6.07	0.62	0.61	1.45	2.01	5.11	6.21	0.12
DG(16:0_18:1)	1.21	1.74	0.80	< LOD	0.87	2.00	1.05	0.52	0.53	1.13	1.97	2.69	0.81	0.93	1.28	1.42	4.18	2.23	0.23	1.18	0.51	1.42	0.94	3.26	0.72	0.66	1.10	0.67	< LOD	2.33	0.83	< LOD	2.48	1.01	1.21	2.55	3.00	1.34	0.83	2.26	1.82	1.09	1.85	1.56	2.28	1.95	2.89
Hex2Cer(d18:1/16:0)	0.32	0.72	0.29	0.46	0.42	0.42	0.44	0.40	0.54	0.62	0.74	0.57	0.45	0.44	0.35	0.36	0.47	0.50	0.38	0.72	0.72	0.38	0.54	0.42	0.66	0.54	0.63	0.58	0.35	0.56	0.34	0.32	0.41	0.44	0.44	0.74	0.68	0.76	0.60	0.50	0.28	0.42	0.51	0.67	0.77	0.70	0.74
Hex2Cer(d18:1/24:1)	< LOD	0.07	0.04	0.06	0.07	< LOD	0.05	0.05	0.07	< LOD	0.08	0.08	< LOD	0.04	0.06	0.08	0.02	0.03	0.05	0.05	0.09	0.05	0.04	0.07	0.06	0.06	0.08	0.04	0.06	0.04	0.06	0.07	0.05	< LOD	0.05	0.06	0.08	0.08	0.08	0.07	0.06	0.05	0.08	0.07	0.06	0.08	0.08
Docosahexaenoid acid (FA(22:6ω3))	0.85	1.89	1.64	2.11	1.50	1.01	0.67	0.47	1.74	0.59	1.99	1.23	0.90	2.16	< LOD	1.55	1.40	1.36	1.50	1.23	1.33	1.44	1.25	2.70	1.07	1.78	0.96	0.76	0.64	1.35	0.66	0.54	0.81	1.36	0.47	0.76	1.28	1.88	2.34	1.82	1.44	2.85	1.16	1.42	1.81	0.70	1.08
Eicosapentaenoic acid (FA(20:5ω3))	0.14	0.36	0.29	0.35	0.26	0.21	0.11	< LOD	0.27	0.15	0.44	0.19	0.17	0.54	< LOD	< LOD	0.17	0.18	0.21	0.38	0.25	0.15	0.24	0.54	0.34	0.38	0.17	0.10	0.09	0.29	0.13	< LOD	0.18	0.20	< LOD	0.09	0.20	0.28	0.36	0.31	0.25	0.30	0.17	0.17	0.30	0.20	0.14
Octadecenoic acid	47.49	65.43	62.25	83.93	119.12	86.25	55.01	< LOD	70.99	45.04	159.86	70.53	< LOD	115.44	91.24	196.52	174.75	148.28	< LOD	< LOD	< LOD	110.17	< LOD	141.08	< LOD	71.31	82.12	29.73	78.27	81.13	28.31	< LOD	78.27	62.10	30.74	62.44	76.72	54.58	93.03	95.75	148.53	138.98	103.19	62.38	149.57	109.92	72.76
HexCer(d18:1/16:0)	0.14	0.31	0.08	0.14	0.22	0.14	0.17	0.17	0.20	0.24	0.35	0.22	0.18	0.14	0.17	0.16	0.23	0.22	0.12	0.38	0.20	0.30	0.29	0.25	0.26	0.31	0.24	0.19	0.13	0.21	0.10	0.15	0.22	0.21	0.19	0.24	0.26	0.19	0.25	0.22	0.12	0.22	0.30	0.37	0.26	0.28	0.31
HexCer(d18:1/18:0)	0.04	0.05	< LOD	< LOD	0.05	0.04	< LOD	0.04	0.05	0.05	0.08	0.04	< LOD	< LOD	0.05	0.06	0.04	0.04	0.05	0.06	0.04	0.08	0.04	0.06	0.04	0.05	0.04	< LOD	< LOD	0.05	< LOD	< LOD	< LOD	< LOD	0.03	0.05	< LOD	0.05	0.05	0.05	< LOD	0.09	0.05	0.05	0.05	0.10	0.08
HexCer(d18:1/22:0)	< LOD	0.35	< LOD	0.21	0.33	0.23	0.25	< LOD	0.32	0.32	0.33	0.24	< LOD	< LOD	0.23	0.13	0.21	0.23	0.25	0.42	0.24	0.32	0.33	0.23	0.23	0.28	0.27	0.40	0.33	0.24	< LOD	0.28	0.39	0.34	0.22	0.25	0.36	0.38	0.31	0.25	< LOD	0.25	0.48	0.42	0.36	0.31	0.29
Indoxyl sulfate	0.34	0.14	1.01	1.01	1.18	0.14	0.71	0.36	0.11	0.15	0.73	0.45	0.40	< LOD	1.01	0.54	0.65	0.56	1.31	0.47	0.87	0.63	0.34	0.42	0.73	0.20	< LOD	0.17	0.68	0.10	1.16	1.20	0.22	0.19	0.60	0.32	< LOD	0.46	1.12	1.10	1.11	0.26	0.13	0.34	0.32	0.33	< LOD
lysoPC a C14:0	7.10	6.41	< LOD	3.19	3.39	3.68	7.82	2.69	2.70	6.85	12.91	9.37	5.99	4.63	4.18	2.29	9.58	8.12	4.03	5.31	< LOD	8.06	6.18	7.73	7.17	9.71	5.33	2.99	2.33	5.18	3.58	< LOD	7.63	4.65	4.31	4.96	5.17	4.41	4.58	10.62	4.12	8.10	6.10	3.80	6.84	4.50	5.31
lysoPC a C16:0	9.20	21.23	4.08	8.50	16.00	10.89	19.11	10.23	17.47	30.29	31.32	24.25	13.78	21.99	10.52	5.12	17.72	17.70	12.19	24.93	5.26	14.21	30.53	16.51	26.66	21.75	14.30	17.17	6.87	15.93	8.09	4.11	15.44	20.12	8.31	23.97	23.31	19.95	22.08	25.35	8.38	17.57	27.86	22.30	27.43	17.86	16.81
lysoPC a C16:1	1.92	1.92	0.78	1.59	2.73	2.05	3.47	1.64	0.93	2.21	6.69	3.59	2.33	1.45	2.29	1.16	4.70	4.55	2.55	2.25	0.61	4.41	1.91	4.82	3.34	4.95	4.05	1.57	1.25	2.91	1.42	0.70	4.03	0.94	1.63	1.09	1.60	2.12	1.45	5.00	1.57	3.98	1.15	1.12	0.98	1.61	2.96
lysoPC a C17:0	0.46	0.61	0.26	0.40	0.62	0.47	0.81	0.58	0.35	1.29	1.12	0.82	0.65	0.83	0.39	0.23	0.81	0.83	0.49	1.34	0.30	0.58	0.71	0.74	1.22	0.99	0.68	0.52	0.28	1.03	0.36	0.22	0.60	0.62	0.29	0.57	0.83	0.78	0.54	0.77	0.36	0.70	0.61	0.43	0.55	0.79	0.94
lysoPC a C18:0	5.05	11.31	2.02	4.26	10.71	6.95	12.28	8.53	11.35	28.09	15.00	13.21	7.96	16.02	5.55	2.67	9.78	11.69	5.68	22.68	4.38	4.62	19.84	9.13	18.73	14.33	10.53	10.36	3.51	13.45	3.27	2.18	7.68	13.57	4.04	19.15	18.04	12.75	14.27	14.38	4.92	6.27	17.46	13.71	18.08	16.51	13.75
lysoPC a C18:1	15.77	22.97	4.29	13.16	22.96	20.95	27.70	18.25	10.80	30.45	42.36	33.89	19.36	19.07	10.24	4.81	35.49	41.39	13.33	37.06	4.45	19.62	25.31	44.66	42.17	43.34	37.29	14.24	5.77	33.58	6.55	3.52	26.17	19.15	8.38	21.20	28.22	22.44	16.89	39.81	9.72	22.41	28.43	17.50	25.11	25.51	33.13
lysoPC a C18:2	12.83	48.12	< LOD	10.16	15.64	12.57	15.50	8.73	12.26	24.44	27.09	18.23	8.28	22.12	3.75	1.97	21.05	26.48	3.08	47.67	2.44	6.86	48.41	17.84	46.82	26.27	32.27	13.11	< LOD	30.26	2.53	< LOD	11.42	39.05	3.75	43.80	50.14	13.07	29.22	24.96	5.65	8.60	75.13	34.69	75.27	47.38	23.15
lysoPC a C20:3	0.85	1.25	0.25	0.63	1.46	1.35	1.52	0.56	0.75	1.59	2.02	2.16	1.35	1.07	0.61	0.48	2.04	2.58	0.71	2.22	0.28	1.21	1.61	2.38	3.74	2.20	2.04	1.01	0.44	1.75	0.39	0.31	1.39	1.05	0.50	1.43	1.51	1.76	1.11	3.15	0.75	1.63	1.56	1.08	1.39	2.21	1.35
lysoPC a C20:4	1.23	2.32	0.33	1.09	2.71	1.82	2.39	0.76	0.96	2.10	3.56	2.59	1.76	1.78	0.63	0.47	1.85	3.19	0.76	3.33	0.36	1.79	2.36	2.15	6.59	3.37	2.98	2.07	0.62	2.24	0.41	0.48	1.85	1.46	0.53	2.06	2.43	2.26	2.13	3.77	0.63	1.77	2.99	1.96	2.50	2.64	1.37
lysoPC a C28:1	< LOD	0.14	< LOD	< LOD	< LOD	< LOD	0.32	0.10	0.09	0.24	0.36	0.20	0.20	0.23	< LOD	< LOD	0.18	0.23	0.17	0.25	< LOD	0.16	0.18	0.17	0.38	0.18	< LOD	0.30	< LOD	0.32	< LOD	< LOD	0.25	0.16	< LOD	0.26	0.24	0.19	0.13	0.21	< LOD	< LOD	0.27	0.10	0.17	0.17	0.18
PC aa C28:1	0.63	1.18	0.22	0.62	0.53	0.44	1.15	0.97	0.79	1.57	1.63	1.09	0.67	0.94	0.65	0.59	1.45	1.24	0.53	1.82	0.88	0.54	1.36	0.95	2.13	1.04	1.14	1.21	0.34	1.32	0.40	0.33	1.03	1.17	0.49	2.19	1.39	1.08	1.25	1.26	0.41	0.43	1.90	1.52	1.84	2.03	1.71
PC aa C30:0	2.96	4.16	1.82	2.16	2.06	1.71	3.35	2.54	2.63	3.95	6.46	4.54	2.39	2.92	3.10	3.39	6.02	4.63	1.87	5.25	2.31	3.91	3.21	4.29	4.88	3.44	2.94	3.04	2.15	3.66	2.82	1.34	4.38	3.15	2.56	4.98	4.74	5.08	4.15	5.60	2.10	3.91	5.00	4.16	5.98	5.72	5.00
PC aa C32:0	2.91	7.34	3.57	3.84	3.72	2.68	4.93	3.12	6.17	4.99	11.06	6.41	3.24	4.69	4.39	5.72	9.05	6.51	4.32	8.49	4.83	9.37	7.61	6.10	8.31	4.97	4.34	5.78	3.92	4.05	3.22	3.35	5.57	4.39	3.71	9.00	6.95	6.99	5.97	6.70	2.66	6.43	8.22	9.39	8.87	7.32	7.28
PC aa C32:1	5.97	7.02	6.03	4.60	4.06	3.93	9.35	4.87	3.22	5.45	17.49	12.12	6.42	3.44	15.44	16.21	19.46	12.80	9.75	8.11	5.50	17.60	5.05	17.95	10.31	9.19	7.36	4.16	7.87	6.66	8.75	5.61	14.94	3.31	11.73	7.34	5.28	7.43	3.94	12.64	6.19	12.59	4.96	5.75	4.64	6.57	11.61
PC aa C32:2	2.14	3.97	0.92	0.99	0.88	1.00	1.96	1.72	0.57	1.59	4.50	3.18	1.30	1.31	1.20	1.26	2.35	1.83	0.72	2.22	0.83	1.40	1.22	1.77	2.14	1.50	1.33	0.83	0.97	1.96	1.60	0.63	2.97	2.29	2.09	4.36	3.22	1.49	1.66	2.37	1.17	1.74	3.66	1.82	3.54	3.24	3.33
PC aa C32:3	0.39	1.27	0.08	0.28	0.21	0.26	0.27	0.41	0.44	0.51	0.63	0.44	0.19	0.52	0.19	0.14	0.69	0.61	0.16	1.60	0.52	0.20	0.68	0.35	1.16	0.54	0.67	0.30	0.15	0.78	0.14	0.06	0.32	0.91	0.19	1.45	1.10	0.47	1.33	0.56	0.18	0.19	1.62	1.08	1.71	2.13	1.11
PC aa C34:1	46.35	78.44	59.33	40.32	46.52	44.19	83.09	66.78	47.41	81.07	124.72	118.29	61.03	49.34	113.57	108.27	158.17	111.40	98.65	109.46	59.06	125.31	66.60	143.29	104.10	82.65	62.64	55.73	70.91	66.43	66.42	67.47	111.39	46.97	87.45	105.11	78.76	90.27	52.61	106.41	53.85	97.61	84.91	83.16	78.44	86.60	144.44
PC aa C34:2	37.57	138.48	14.93	28.10	26.05	24.53	49.46	30.16	56.45	67.11	77.66	70.35	29.24	53.28	45.32	44.93	91.95	72.68	25.29	130.55	33.84	37.19	127.90	61.00	117.06	49.62	50.75	43.05	20.50	58.70	26.54	14.18	53.93	93.81	40.15	210.36	128.80	50.90	82.66	72.41	28.67	37.99	200.15	155.73	186.01	139.42	98.23
PC aa C34:3	6.76	6.06	2.66	4.32	3.08	3.41	7.30	4.62	2.19	5.07	12.12	10.62	3.74	2.91	5.09	4.96	12.13	9.23	3.26	12.09	4.50	5.16	4.47	11.61	13.30	8.26	7.59	2.68	2.42	9.59	4.53	1.71	9.18	3.07	6.39	4.95	5.96	5.72	3.44	10.45	4.35	5.19	4.54	4.38	3.79	8.98	13.58
PC aa C34:4	0.43	0.52	0.22	0.30	0.26	0.31	0.51	0.26	0.16	0.44	1.07	0.76	0.31	0.28	0.39	0.38	0.95	0.79	0.26	0.84	0.34	0.51	0.30	0.68	1.11	0.61	0.54	0.23	0.23	0.59	0.37	0.17	0.77	0.31	0.45	0.63	0.48	0.51	0.36	0.68	0.31	0.41	0.55	0.43	0.45	0.82	0.68
PC aa C36:0	0.56	1.12	0.25	0.79	0.55	0.43	0.78	0.69	0.65	0.73	0.93	0.79	0.68	1.25	0.20	< LOD	0.65	0.93	0.39	2.04	0.37	0.37	0.90	0.64	1.30	1.26	0.77	1.01	0.32	0.63	0.56	0.20	0.94	0.93	0.27	1.02	1.29	1.51	1.22	0.96	0.35	0.49	0.84	0.80	1.18	0.84	1.37
PC aa C36:1	40.16	50.17	41.34	35.02	41.25	43.35	83.78	79.26	32.86	94.30	83.98	90.99	54.91	40.55	88.33	72.70	132.93	100.29	69.00	117.97	57.06	73.36	51.67	118.86	86.47	76.48	70.23	41.49	53.56	85.13	46.63	49.90	92.23	40.02	59.73	85.89	78.83	83.13	36.68	92.59	49.38	65.05	51.30	59.15	51.13	99.36	163.93
PC aa C36:2	53.58	139.62	31.68	44.62	44.47	46.82	83.26	66.21	65.21	113.68	101.08	91.87	52.45	73.61	66.53	58.25	130.74	111.84	51.27	208.49	68.96	57.44	146.19	100.96	160.14	93.07	100.34	57.23	33.37	118.09	33.45	32.45	86.00	107.87	48.64	262.52	174.56	77.54	97.10	100.25	42.35	53.32	211.04	175.05	216.50	205.99	178.51
PC aa C36:3	28.05	51.53	13.61	21.08	19.19	21.49	36.72	22.68	25.55	41.55	53.95	49.33	26.62	29.13	25.65	24.40	54.41	47.94	17.79	83.56	29.03	24.88	47.58	42.50	76.43	41.29	41.35	22.68	15.90	52.93	17.52	14.25	39.94	35.16	24.38	68.42	61.02	36.25	30.81	49.46	19.75	28.01	58.21	51.28	56.21	72.90	70.87
PC aa C36:4	10.35	17.06	6.45	6.91	9.24	9.02	15.35	6.42	7.71	14.51	25.33	21.45	10.89	9.96	12.87	14.14	23.62	22.06	11.11	24.93	11.27	16.99	14.30	15.75	36.47	16.98	13.19	11.04	10.86	15.15	8.56	9.25	18.04	9.94	10.62	21.42	16.15	18.01	11.74	21.51	7.89	13.79	24.26	19.47	17.93	24.32	20.01
PC aa C36:5	3.51	4.18	2.58	2.58	2.05	2.25	3.71	1.98	1.81	3.38	6.35	6.82	3.03	2.68	1.88	1.93	4.21	4.02	3.91	9.42	3.01	2.41	3.44	6.09	11.89	6.16	2.63	1.64	1.36	4.78	3.33	1.53	4.91	2.10	2.34	2.70	3.77	4.89	2.75	6.68	2.67	4.12	2.47	2.74	2.76	4.61	4.63
PC aa C36:6	0.20	0.43	0.15	0.17	0.14	0.12	0.25	0.18	0.18	0.22	0.43	0.34	0.17	0.24	0.13	0.15	0.23	0.22	0.18	0.62	0.16	0.16	0.22	0.30	0.49	0.31	0.25	0.21	0.11	0.30	0.14	0.08	0.29	0.24	0.11	0.36	0.31	0.33	0.43	0.33	0.11	0.19	0.25	0.22	0.27	0.40	0.28
PC aa C38:0	0.24	0.56	0.20	0.35	0.28	0.21	0.38	0.38	0.32	0.34	0.49	0.52	0.41	0.43	0.27	0.28	0.45	0.32	0.47	0.83	0.23	0.37	0.45	0.40	0.51	0.44	0.43	0.49	0.25	0.38	0.27	0.23	0.38	0.35	0.23	0.55	0.60	0.60	0.52	0.47	0.22	0.36	0.50	0.54	0.54	0.39	0.56
PC aa C38:1	0.10	0.11	0.07	0.13	0.18	0.11	0.22	0.18	0.12	0.26	0.19	0.19	0.13	0.26	0.39	0.59	1.57	1.44	0.97	2.83	0.87	0.58	1.84	1.06	1.79	1.66	1.11	0.14	0.08	0.25	0.11	0.07	0.22	0.21	0.08	0.29	0.40	0.29	0.20	0.38	0.08	0.22	0.21	0.35	0.27	0.39	0.59
PC aa C38:3	5.08	7.99	6.43	5.18	8.00	6.16	11.00	5.96	8.68	13.30	9.90	12.55	9.59	8.04	12.95	12.22	16.02	13.68	10.20	17.10	9.35	13.22	10.04	10.33	16.75	9.52	8.44	11.88	13.24	10.03	6.97	8.71	11.48	7.81	8.04	19.06	11.24	16.28	8.44	14.48	8.27	12.16	10.05	12.66	11.16	16.70	14.31
PC aa C38:4	8.33	11.08	7.71	7.43	11.29	9.32	17.74	7.19	9.39	16.09	18.34	16.27	12.73	10.77	14.22	15.46	20.58	20.78	11.15	25.68	13.25	19.08	12.21	12.74	32.87	15.11	13.75	15.98	14.72	14.58	7.60	12.10	16.65	8.36	8.55	22.99	14.85	20.86	12.63	19.43	7.72	13.71	15.73	17.88	16.84	25.70	18.87
PC aa C38:5	7.52	10.52	7.28	7.02	7.35	6.28	10.42	5.99	7.77	10.09	15.12	14.52	9.57	9.59	6.79	7.85	11.19	11.66	10.73	24.83	9.77	10.50	10.42	12.90	26.64	14.41	8.39	8.60	7.43	11.84	7.52	7.05	11.88	7.10	5.35	10.80	10.65	15.16	10.04	15.16	5.90	11.77	7.79	9.57	9.68	14.10	13.26
PC aa C38:6	2.53	4.39	2.61	2.73	2.01	1.77	3.68	2.03	3.16	2.52	4.33	5.71	3.92	3.29	1.89	2.48	2.82	3.19	4.57	6.66	2.63	3.57	3.69	4.62	7.32	5.21	2.23	2.68	2.54	3.76	2.67	2.15	4.02	2.51	1.86	3.51	3.54	5.59	3.51	6.06	2.04	5.24	2.90	3.34	3.73	3.41	4.01
PC aa C40:2	0.06	0.08	< LOD	< LOD	0.06	0.06	0.09	0.06	0.06	0.04	0.08	0.10	0.06	0.06	0.07	0.07	0.11	0.11	0.10	0.10	0.06	0.09	0.10	0.05	0.12	0.07	0.07	0.07	< LOD	0.09	0.04	0.06	0.10	0.06	0.05	0.12	0.09	0.08	0.06	0.07	0.05	0.06	0.06	0.06	0.09	0.08	0.09
PC aa C40:3	0.09	0.20	0.08	0.16	0.17	0.14	0.23	0.16	0.20	0.24	0.19	0.17	0.18	0.25	0.14	0.15	0.33	0.28	0.17	0.52	0.19	0.19	0.29	0.19	0.41	0.24	0.23	0.26	0.13	0.32	0.12	0.12	0.25	0.24	0.12	0.40	0.39	0.29	0.24	0.22	0.15	0.15	0.26	0.31	0.29	0.32	0.31
PC aa C40:4	0.44	0.84	0.35	0.61	0.87	0.50	1.22	0.55	1.00	1.35	1.02	0.80	0.60	1.20	1.07	1.01	1.39	1.15	0.59	1.69	0.70	0.84	1.25	0.51	1.93	0.68	0.83	1.65	0.95	1.14	0.38	0.58	1.00	1.07	0.48	2.24	1.73	1.64	1.07	0.95	0.38	0.59	1.41	1.90	1.14	1.95	1.07
PC aa C40:5	1.89	3.49	2.24	2.12	2.82	1.64	3.40	2.67	4.54	4.14	3.81	4.99	3.33	4.60	3.48	3.54	3.12	2.82	4.48	9.03	3.05	3.53	5.67	4.20	6.24	3.60	2.56	4.65	3.97	3.17	2.78	2.66	3.61	3.84	2.05	5.93	5.42	6.37	5.09	4.57	2.12	5.00	3.78	5.11	5.24	6.19	4.61
PC aa C40:6	2.71	4.33	3.63	3.15	2.35	1.64	4.56	2.90	4.43	3.12	3.51	5.95	4.76	4.83	4.09	4.27	3.15	3.12	7.40	6.68	2.80	5.10	4.89	5.03	4.96	4.79	2.14	3.89	4.77	3.83	3.90	3.67	4.48	3.71	2.52	4.66	5.44	8.30	4.50	6.83	2.87	7.73	2.80	4.14	5.74	3.82	4.75
PC aa C42:0	0.05	0.07	< LOD	< LOD	0.05	< LOD	0.06	< LOD	< LOD	0.06	0.06	0.06	0.06	0.07	0.05	0.03	0.09	0.09	0.04	0.14	0.03	0.05	0.12	0.08	0.14	0.08	0.08	0.05	< LOD	0.08	< LOD	< LOD	0.07	0.09	0.05	0.10	0.08	0.07	0.07	0.06	< LOD	0.05	0.14	0.08	0.12	0.10	0.09
PC aa C42:1	0.02	0.05	0.02	0.03	0.03	0.03	0.05	0.03	0.02	0.04	0.06	0.06	0.04	0.03	0.06	0.05	0.05	0.06	0.05	0.07	0.03	0.04	0.06	0.04	0.06	0.05	0.05	0.03	0.03	0.03	0.02	< LOD	0.06	0.03	0.04	0.06	0.06	0.07	0.05	0.06	0.03	0.05	0.06	0.06	0.06	0.05	0.06
PC aa C42:4	0.04	0.03	0.03	0.03	0.03	0.03	0.06	< LOD	0.04	0.02	0.06	0.06	0.04	0.03	0.04	0.03	0.04	0.05	0.04	0.05	0.03	0.05	0.05	0.05	0.06	0.04	0.04	0.04	0.04	0.03	0.03	0.02	0.06	< LOD	0.04	0.07	0.04	0.07	0.04	0.04	< LOD	0.03	0.06	0.04	0.05	0.05	0.05
PC aa C42:5	0.09	0.19	0.07	0.15	0.23	0.06	0.14	0.14	0.26	0.16	0.13	0.19	0.11	0.36	0.12	0.10	0.11	0.11	0.10	0.71	0.22	0.08	0.35	0.16	0.29	0.15	0.14	0.23	0.12	0.19	0.09	0.08	0.16	0.28	0.09	0.23	0.28	0.26	0.35	0.18	0.08	0.11	0.22	0.27	0.22	0.28	0.22
PC aa C42:6	< LOD	0.22	< LOD	0.22	0.24	< LOD	0.34	< LOD	0.26	0.30	< LOD	0.22	< LOD	0.32	0.13	0.12	0.22	0.23	0.26	0.44	0.14	0.14	0.35	0.19	0.37	0.29	0.16	0.27	< LOD	0.26	< LOD	< LOD	0.30	0.26	< LOD	0.38	0.35	0.39	0.24	0.28	< LOD	< LOD	0.25	0.37	0.21	0.27	< LOD
PC ae C30:0	0.44	0.36	0.17	0.28	0.23	0.22	0.49	0.34	0.31	0.66	0.92	0.54	0.31	0.32	0.52	0.40	0.71	0.67	0.43	0.91	0.41	0.49	0.42	0.74	0.89	0.54	0.56	0.52	0.26	0.46	0.30	0.21	0.54	0.30	0.34	0.65	0.54	0.47	0.53	0.61	0.22	0.28	0.55	0.40	0.52	0.61	0.44
PC ae C30:1	0.19	0.20	< LOD	0.11	0.10	0.07	0.17	0.10	0.15	< LOD	0.39	0.20	0.17	0.15	0.16	0.13	0.23	0.22	0.15	0.39	0.16	0.20	0.12	0.22	0.36	0.23	0.22	0.25	0.09	0.33	0.11	0.06	0.22	0.07	0.10	0.26	0.35	0.25	< LOD	< LOD	< LOD	< LOD	0.09	< LOD	< LOD	0.07	< LOD
PC ae C30:2	0.02	0.03	0.02	0.03	0.02	0.02	0.04	0.03	0.02	0.05	0.07	0.04	0.05	0.04	0.04	0.04	0.06	0.06	0.04	0.10	0.05	0.05	0.05	0.05	0.09	0.06	0.06	0.06	0.03	0.05	0.02	0.02	0.04	0.03	0.03	0.05	0.05	0.06	0.04	0.04	0.03	0.03	0.07	0.04	0.05	0.08	0.07
PC ae C32:1	0.90	1.67	0.72	1.26	0.95	0.64	1.36	1.19	1.42	1.73	2.51	1.48	0.79	1.18	1.49	1.47	1.76	1.51	1.29	2.55	1.23	1.76	1.65	1.91	2.25	1.53	1.78	1.73	0.83	1.25	0.76	0.78	1.35	1.12	0.86	2.48	1.79	1.54	1.98	1.56	0.73	1.03	2.35	2.30	2.18	2.00	1.90
PC ae C32:2	0.49	0.97	0.36	0.64	0.50	0.45	0.68	0.85	0.53	0.87	1.37	0.82	0.50	0.61	0.55	0.57	0.93	0.81	0.60	1.54	0.68	0.64	0.72	0.75	1.28	0.85	1.05	0.62	0.46	0.89	0.38	0.38	0.73	0.74	0.40	1.13	1.12	0.90	0.85	0.81	0.36	0.53	1.19	0.97	0.97	1.18	1.19
PC ae C34:0	0.51	0.51	0.55	0.60	0.47	0.39	0.72	0.44	0.35	0.66	1.25	0.86	0.47	0.44	0.54	0.58	1.36	0.91	0.52	1.15	0.68	1.07	0.49	0.80	1.05	0.76	0.69	0.59	0.44	0.72	0.38	0.43	0.76	0.47	0.52	0.67	0.64	0.86	0.44	0.78	0.29	0.75	0.50	0.56	0.52	0.82	1.09
PC ae C34:1	4.50	6.08	4.23	4.73	4.63	3.05	6.48	6.06	4.30	6.61	9.44	7.99	4.15	3.97	7.48	7.37	11.65	8.67	6.37	11.66	6.08	9.89	6.06	10.90	11.37	7.40	7.15	6.46	4.60	6.60	4.61	3.82	7.64	3.75	5.42	6.43	6.83	6.82	5.37	7.08	3.73	6.36	5.87	6.74	6.00	7.52	11.53
PC ae C34:2	2.59	4.87	1.48	3.23	2.40	2.17	3.37	3.63	2.91	4.41	6.59	4.60	2.28	2.83	3.26	3.57	5.72	4.90	3.57	7.40	3.69	3.53	4.50	4.75	7.10	4.89	5.36	2.91	1.94	3.91	1.78	1.77	3.41	3.34	2.36	5.69	5.34	4.12	4.16	4.13	1.95	2.81	6.22	4.98	5.83	5.91	6.23
PC ae C34:3	1.01	3.83	0.27	1.47	0.99	1.02	1.33	1.51	1.80	1.91	2.86	1.57	0.71	1.69	0.59	0.64	2.35	1.93	0.62	3.74	1.64	0.63	2.75	0.98	3.28	1.75	2.86	1.34	0.43	1.97	0.40	0.33	1.10	2.79	0.55	5.68	3.27	1.83	3.79	1.46	0.47	0.60	6.76	4.16	5.54	3.98	2.79
PC ae C36:0	0.27	0.69	0.25	0.34	0.36	0.25	0.44	0.45	0.40	0.45	0.59	0.58	0.28	0.44	0.69	0.66	0.79	0.69	0.59	1.42	0.61	0.62	0.70	0.52	0.97	0.59	0.64	0.43	0.25	0.41	0.28	0.23	0.39	0.47	0.29	0.59	0.57	0.67	0.72	0.69	0.29	0.33	0.62	0.59	0.77	0.83	0.83
PC ae C36:1	3.59	2.86	4.25	3.03	2.67	2.62	5.58	5.21	1.58	5.67	6.44	5.80	4.01	2.78	7.09	6.23	11.78	8.06	6.19	9.72	5.26	7.64	3.01	10.57	8.31	6.69	5.47	2.74	4.06	6.54	3.53	3.72	6.64	2.14	3.89	3.37	4.47	5.06	2.04	5.55	3.18	5.64	2.54	2.66	2.43	6.01	11.99
PC ae C36:2	3.71	6.31	2.79	3.48	2.72	2.52	4.99	4.11	2.41	5.28	6.77	5.63	3.52	3.67	4.86	4.57	9.27	7.61	4.10	12.73	4.94	5.44	5.63	7.96	11.65	6.48	6.03	3.11	2.52	7.14	2.61	2.22	5.72	4.16	3.17	7.55	7.88	4.93	3.66	5.38	2.78	4.09	6.64	5.58	5.89	9.06	10.76
PC ae C36:3	1.67	2.66	0.91	1.66	1.18	1.17	1.97	1.75	1.35	2.11	3.17	2.72	1.48	1.41	1.44	1.38	3.06	2.82	1.29	4.22	2.08	1.71	2.08	2.84	4.51	2.55	2.71	1.55	0.94	2.93	1.00	0.75	2.14	1.58	1.24	2.59	2.53	2.34	1.98	2.21	1.04	1.43	2.49	2.42	2.47	2.81	3.68
PC ae C36:4	0.81	1.90	0.49	1.05	1.17	0.86	1.38	1.01	1.21	1.88	2.19	1.49	0.81	1.01	0.86	0.98	1.79	1.64	0.92	2.92	1.37	1.35	1.92	1.14	3.18	1.38	2.20	2.04	0.70	1.36	0.53	0.64	1.18	1.24	0.72	2.85	2.09	2.03	2.13	1.39	0.56	0.79	3.26	2.43	2.59	2.37	1.79
PC ae C36:5	0.64	1.47	0.49	0.89	0.99	0.59	1.08	0.76	1.04	1.47	1.93	1.24	0.73	0.96	0.67	0.99	1.18	1.28	0.91	2.53	1.13	1.32	1.46	0.76	2.70	1.27	1.67	1.52	0.77	0.99	0.47	0.65	0.80	1.08	0.53	2.32	1.58	1.74	1.67	1.14	0.40	0.65	2.60	2.18	1.91	1.94	1.21
PC ae C38:0	0.34	0.61	< LOD	0.35	0.32	< LOD	0.38	0.32	0.37	0.44	0.69	0.59	0.40	0.53	0.30	0.31	0.42	0.47	0.44	1.01	0.38	0.33	0.53	0.51	0.96	0.62	0.42	0.44	< LOD	0.54	< LOD	< LOD	0.48	0.45	< LOD	0.48	0.62	0.62	0.53	0.57	< LOD	0.38	0.44	0.46	0.54	0.59	0.56
PC ae C38:1	0.26	0.30	0.30	0.30	0.27	0.30	0.71	0.39	0.21	0.60	0.48	0.59	0.38	0.39	0.73	0.64	1.37	0.87	0.67	1.26	0.57	0.71	0.45	0.89	0.86	0.62	0.55	0.29	0.44	0.65	0.32	0.31	0.60	0.34	0.28	0.47	0.65	0.54	0.12	0.52	0.30	0.55	0.42	0.35	0.22	0.66	1.14
PC ae C38:2	0.73	0.93	0.64	0.61	0.60	0.51	1.11	0.68	0.59	1.24	1.21	1.15	0.94	0.86	1.24	1.10	2.25	1.68	1.26	2.17	0.95	1.10	1.15	1.32	1.93	1.22	1.10	0.67	0.75	1.37	0.59	0.54	1.37	0.82	0.65	1.40	1.45	1.26	0.63	1.05	0.60	0.86	0.82	0.99	0.95	1.41	2.05
PC ae C38:3	0.65	0.91	0.79	0.57	0.55	0.50	1.03	0.63	0.57	1.13	1.10	1.16	0.96	0.78	1.10	1.05	1.98	1.67	1.07	2.07	0.99	1.39	1.12	1.32	2.20	1.19	1.00	0.94	0.89	1.33	0.63	0.71	1.25	0.70	0.66	1.25	1.39	1.27	0.74	1.29	0.67	1.17	0.94	1.09	0.91	1.59	1.81
PC ae C38:4	0.77	1.02	0.72	0.72	0.90	0.62	1.31	0.71	0.63	1.43	1.56	1.20	1.02	0.83	1.22	1.21	1.83	1.71	1.11	2.51	1.27	1.84	1.12	1.31	3.24	1.38	1.35	1.52	1.06	1.26	0.60	0.89	1.30	0.74	0.66	1.50	1.34	1.69	0.97	1.26	0.57	1.05	1.41	1.21	1.09	1.78	1.55
PC ae C38:5	0.95	1.69	0.68	1.13	1.32	0.75	1.24	1.19	1.23	1.74	2.08	1.49	0.97	1.12	1.10	1.09	1.41	1.42	1.15	3.87	1.52	1.56	2.03	1.44	3.59	1.52	1.87	2.49	0.82	1.37	0.67	0.78	1.15	1.08	0.72	1.97	1.95	2.25	1.87	1.31	0.57	0.94	2.02	2.01	1.92	1.91	1.73
PC ae C38:6	0.66	1.83	0.50	0.89	0.89	0.53	0.82	1.03	1.01	1.18	1.68	1.22	0.77	0.97	0.68	0.72	1.03	0.80	0.90	2.43	0.79	0.84	1.41	0.94	1.83	1.29	1.51	1.44	0.55	0.79	0.58	0.49	0.65	1.09	0.45	1.81	1.46	1.58	2.00	1.07	0.39	0.65	1.67	1.67	1.73	1.49	1.29
PC ae C40:1	0.09	0.16	0.10	0.14	0.07	0.08	0.16	0.09	0.08	0.12	0.30	0.23	0.15	0.17	0.10	0.12	0.15	0.15	0.18	0.25	0.14	0.20	0.16	0.21	0.37	0.21	0.12	0.12	0.09	0.17	0.09	0.08	0.19	0.13	0.05	0.13	0.17	0.20	0.12	0.23	0.09	0.15	0.17	0.16	0.16	0.12	0.17
PC ae C40:2	0.17	0.30	0.14	0.23	0.26	0.14	0.26	0.30	0.27	0.35	0.37	0.31	0.21	0.34	0.21	0.20	0.43	0.31	0.25	0.57	0.32	0.25	0.39	0.32	0.49	0.31	0.32	0.30	0.18	0.36	0.14	0.13	0.26	0.31	0.15	0.34	0.41	0.37	0.35	0.36	0.16	0.19	0.32	0.39	0.38	0.42	0.48
PC ae C40:3	0.15	0.31	0.18	0.17	0.20	0.15	0.28	0.20	0.21	0.32	0.33	0.36	0.24	0.27	0.25	0.26	0.38	0.33	0.26	0.44	0.22	0.33	0.32	0.31	0.43	0.23	0.21	0.30	0.28	0.27	0.19	0.21	0.33	0.22	0.21	0.47	0.33	0.43	0.19	0.30	0.18	0.26	0.24	0.26	0.24	0.31	0.34
PC ae C40:4	0.16	0.27	0.22	0.20	0.20	0.17	0.36	0.20	0.22	0.35	0.33	0.32	0.30	0.26	0.42	0.40	0.52	0.40	0.37	0.52	0.33	0.51	0.33	0.33	0.60	0.33	0.32	0.37	0.31	0.29	0.19	0.23	0.38	0.25	0.19	0.50	0.43	0.46	0.23	0.32	0.19	0.31	0.30	0.31	0.30	0.39	0.35
PC ae C40:5	0.49	0.57	0.45	0.55	0.68	0.49	0.57	0.53	0.48	0.58	1.17	0.96	0.76	0.61	1.11	1.17	1.12	0.97	1.01	1.49	1.22	0.87	0.67	0.78	1.25	0.55	0.98	0.97	0.70	0.55	0.51	0.63	0.96	0.57	0.43	0.72	0.76	0.95	0.61	0.52	0.47	0.66	0.53	0.50	0.58	0.66	0.94
PC ae C40:6	0.42	0.67	0.41	0.55	0.43	0.27	0.44	0.56	0.40	0.45	0.64	0.65	0.53	0.58	0.42	0.43	0.51	0.46	0.86	1.34	0.46	0.55	0.71	0.71	0.79	0.57	0.59	0.69	0.38	0.51	0.46	0.34	0.51	0.46	0.26	0.62	0.69	0.88	0.68	0.51	0.30	0.57	0.46	0.52	0.60	0.47	0.68
PC ae C42:2	< LOD	0.14	< LOD	< LOD	< LOD	< LOD	0.14	< LOD	0.08	0.12	0.17	0.14	0.11	0.10	< LOD	0.08	0.09	0.11	0.11	0.16	< LOD	0.11	0.12	0.11	0.18	0.11	< LOD	0.10	0.08	0.12	0.08	< LOD	0.14	< LOD	< LOD	0.15	0.11	0.17	< LOD	0.11	< LOD	0.09	0.08	0.09	< LOD	0.08	0.10
PC ae C42:3	0.07	0.12	0.07	0.07	0.09	0.07	0.13	0.09	0.09	0.12	0.22	0.16	0.10	0.10	0.06	0.08	0.09	0.09	0.10	0.15	0.06	0.08	0.12	0.12	0.15	0.09	0.09	0.07	0.07	0.12	0.07	0.07	0.16	0.08	0.07	0.16	0.18	0.19	0.09	0.14	0.06	0.10	0.12	0.10	0.09	0.11	0.11
PC ae C44:3	0.02	0.03	< LOD	< LOD	0.04	< LOD	0.04	< LOD	0.04	0.05	0.05	0.03	0.03	0.04	< LOD	< LOD	0.04	0.05	0.04	0.06	0.04	< LOD	0.05	< LOD	0.05	0.05	< LOD	0.03	< LOD	0.06	< LOD	< LOD	0.04	0.03	< LOD	0.05	0.05	0.04	0.03	0.03	< LOD	0.02	0.05	0.03	0.04	0.04	0.04

SM (OH) C14:1	1.10	1.78	0.74	1.19	1.03	0.79	1.30	1.55	1.17	2.51	1.94	1.85	1.14	1.81	1.40	1.36	2.16	2.15	1.60	3.80	2.04	1.36	2.10	1.88	3.42	1.89	1.99	1.95	0.95	1.65	0.82	0.90	1.51	1.99	1.19	2.33	2.00	1.69	2.04	2.40	0.94	1.12	2.48	2.20	3.08	3.25	3.48
SM (OH) C16:1	0.51	0.74	0.35	0.65	0.50	0.38	0.49	0.80	0.53	1.12	0.75	0.76	0.48	0.73	0.68	0.69	0.97	0.99	0.81	1.63	1.08	0.71	0.71	0.82	1.52	0.81	1.20	1.17	0.50	0.98	0.37	0.41	0.60	0.80	0.50	0.80	0.89	0.74	0.74	0.94	0.44	0.65	0.87	0.94	1.14	1.56	1.51
SM (OH) C22:1	0.87	2.39	0.50	1.35	1.79	0.61	1.06	1.78	2.23	2.27	1.77	1.32	0.93	3.09	1.07	1.13	2.17	1.88	1.30	3.92	2.04	0.87	3.20	1.45	2.71	1.62	1.92	3.92	0.87	1.90	0.67	0.81	1.20	2.45	0.80	3.31	2.59	2.32	4.25	1.93	0.90	0.95	2.89	3.38	4.84	4.90	4.20
SM (OH) C22:2	0.58	1.04	0.35	0.87	0.84	0.40	0.70	0.97	0.97	1.28	0.90	0.86	0.64	1.44	0.77	0.79	1.29	1.12	1.09	2.11	1.35	0.67	1.43	1.17	1.69	1.06	1.12	1.79	0.56	1.10	0.45	0.54	0.79	1.16	0.54	1.26	1.07	1.06	1.64	1.37	0.58	0.72	1.46	1.59	1.98	2.27	2.04
SM (OH) C24:1	0.07	0.22	0.07	0.13	0.18	0.09	0.10	0.14	0.22	0.17	0.13	0.10	0.08	0.27	0.10	0.13	0.22	0.19	0.13	0.34	0.18	0.10	0.29	0.13	0.19	0.17	0.15	0.43	0.08	0.15	0.07	0.07	0.09	0.22	0.10	0.30	0.25	0.21	0.34	0.17	0.09	0.12	0.27	0.39	0.40	0.47	0.44
SM C16:0	17.61	31.00	10.91	20.13	20.42	14.25	20.02	24.76	25.65	37.25	31.78	27.45	16.06	27.17	23.15	22.85	32.95	35.33	23.93	47.11	32.61	20.47	33.85	28.51	46.97	31.55	37.48	40.69	17.61	27.84	13.89	12.91	23.73	29.23	18.72	47.52	32.94	28.27	38.43	37.76	16.53	21.46	45.07	50.68	60.98	55.50	52.25
SM C16:1	2.11	4.25	1.33	2.62	2.75	1.62	2.80	3.39	3.03	4.61	4.08	3.42	2.17	3.45	2.49	3.01	3.70	4.19	2.54	6.37	3.25	2.26	4.45	3.48	5.63	4.25	4.28	4.95	1.97	3.39	2.15	1.38	2.89	3.69	2.02	5.34	4.91	3.75	5.23	4.69	1.72	2.45	7.43	5.71	8.24	7.04	7.32
SM C18:0	2.14	3.48	1.32	3.06	3.14	1.92	2.33	3.53	3.27	4.31	4.43	3.24	1.74	3.53	3.63	3.88	4.53	4.69	3.93	5.76	4.87	3.91	3.95	4.20	5.70	3.92	5.71	5.79	2.89	4.37	1.53	2.28	2.58	3.24	2.68	4.92	4.04	3.91	4.44	4.15	2.35	3.22	4.70	6.14	5.93	7.89	5.75
SM C18:1	0.80	1.28	0.53	1.07	1.06	0.70	0.93	1.19	1.17	1.72	1.79	1.16	0.85	1.35	1.21	1.51	1.43	1.50	1.37	2.39	1.64	1.22	1.54	1.67	2.24	1.57	1.96	1.96	0.98	1.54	0.62	0.69	0.87	1.34	0.91	2.05	1.54	1.53	1.62	1.71	0.87	1.32	1.99	2.28	2.63	2.94	2.35
SM C20:2	0.04	0.05	< LOD	0.02	0.05	< LOD	0.04	0.02	0.06	0.03	0.02	0.05	0.02	0.02	0.09	0.09	0.18	0.15	0.07	0.19	0.08	0.09	0.08	0.19	0.23	0.15	0.13	0.08	0.04	0.02	0.02	< LOD	0.03	0.05	< LOD	0.07	0.05	0.05	0.07	0.09	0.02	0.04	0.07	0.10	0.08	0.11	0.08
SM C24:0	1.69	4.05	1.53	2.60	3.44	1.41	2.61	2.71	4.58	3.98	3.62	2.86	2.40	5.23	3.09	3.03	5.24	3.78	3.98	6.81	3.70	2.63	5.90	3.55	4.45	3.44	3.32	6.59	2.63	3.32	1.63	2.08	2.98	4.27	2.24	6.17	5.13	5.40	7.24	4.38	2.44	2.87	5.65	6.36	7.53	7.06	7.06
SM C24:1	2.87	4.81	2.21	4.08	4.79	2.09	3.24	4.11	5.60	4.02	5.58	4.57	3.51	5.67	4.57	4.99	5.42	5.22	6.01	6.84	5.66	3.62	6.42	6.21	6.34	6.15	5.14	8.60	3.39	4.52	2.86	3.13	3.79	4.75	3.77	5.20	4.98	5.82	7.45	6.87	2.99	4.04	5.90	7.75	9.39	8.46	9.13
SM C26:0	0.02	0.05	0.03	0.04	0.03	0.02	0.04	0.04	0.05	0.07	0.03	0.03	0.01	0.06	< LOD	0.04	0.05	0.04	0.03	0.10	0.05	0.03	0.08	0.05	0.05	0.06	0.06	0.11	0.03	0.05	< LOD	< LOD	0.02	0.07	0.02	0.09	0.08	0.06	0.10	0.04	0.02	0.01	0.07	0.09	0.09	0.08	0.11
SM C26:1	0.05	0.11	0.06	0.09	0.12	0.02	0.08	0.06	0.13	0.13	0.10	0.06	0.04	0.10	0.06	0.06	0.11	0.06	0.06	0.14	0.09	0.05	0.10	0.09	0.10	0.07	0.10	0.22	0.07	0.08	0.03	0.04	0.05	0.14	0.04	0.18	0.15	0.11	0.25	0.09	0.04	0.04	0.16	0.25	0.21	0.23	0.30
TG(14:0_32:2)	1.42	1.39	0.66	< LOD	< LOD	1.39	0.79	< LOD	< LOD	0.97	1.52	2.57	< LOD	0.61	1.08	1.98	3.65	1.81	0.39	1.02	0.46	0.93	1.59	2.22	0.80	1.18	1.21	< LOD	< LOD	1.64	0.98	< LOD	3.43	1.09	1.77	2.32	2.45	1.19	0.88	2.36	3.19	1.46	1.03	1.44	2.12	1.86	3.31
TG(14:0_34:0)	8.89	3.01	2.40	< LOD	1.46	7.11	4.85	2.14	1.02	2.96	6.62	14.39	2.68	2.01	4.37	8.82	25.34	15.74	1.04	4.02	0.81	2.79	3.46	16.86	2.90	4.02	8.78	1.04	< LOD	9.95	3.78	< LOD	14.91	3.37	10.39	6.78	8.65	6.39	1.22	11.26	9.72	3.75	2.73	3.58	4.91	4.56	18.57
TG(14:0_34:1)	26.67	21.75	12.02	3.66	6.77	27.86	14.41	6.46	4.86	20.01	27.09	56.68	11.20	9.02	17.66	32.51	81.79	48.93	3.86	19.20	3.92	13.04	22.96	57.83	13.47	14.92	24.84	5.34	3.00	34.23	16.32	2.78	59.37	19.20	33.45	52.18	59.59	23.57	9.47	40.33	40.07	18.19	17.24	25.81	35.17	31.02	66.87
TG(14:0_34:2)	5.09	5.75	2.40	< LOD	1.61	5.40	2.68	1.26	1.67	4.06	5.12	9.86	2.52	2.62	2.95	5.25	11.44	6.79	0.74	4.06	1.30	2.60	4.84	9.42	2.90	2.50	3.97	1.77	< LOD	7.33	3.39	< LOD	10.79	4.49	5.79	10.48	11.81	4.85	2.55	6.46	7.34	4.06	5.01	6.75	8.22	8.03	11.66
TG(14:0_36:1)	6.06	2.94	2.21	< LOD	2.27	7.12	3.81	2.12	< LOD	2.94	3.93	9.73	1.98	1.61	2.20	2.77	16.86	11.05	< LOD	3.03	1.12	2.36	2.26	11.24	2.21	2.17	5.48	1.31	< LOD	8.81	2.15	< LOD	11.13	3.25	4.61	6.83	7.90	5.24	1.53	6.22	4.42	2.13	1.97	3.29	4.18	4.47	16.85
TG(14:0_36:2)	10.38	10.70	6.41	2.28	4.03	14.25	6.35	3.59	3.06	8.46	8.35	17.36	3.89	5.37	3.94	6.16	25.52	15.61	1.03	8.42	2.65	5.38	10.22	21.95	4.71	5.17	9.25	2.83	< LOD	18.44	4.77	< LOD	19.29	9.67	6.97	25.89	29.69	9.40	4.75	11.85	9.04	4.95	7.68	11.04	16.26	14.51	25.80
TG(14:0_36:3)	2.61	4.77	1.34	< LOD	< LOD	2.35	1.37	< LOD	1.52	2.79	1.74	4.04	< LOD	2.13	0.79	1.23	3.86	2.43	0.33	2.72	0.64	0.96	3.91	3.63	1.47	1.22	1.51	1.18	< LOD	3.55	1.52	< LOD	3.99	3.99	2.42	8.87	9.78	1.94	2.13	2.15	2.40	1.84	3.31	4.33	5.49	5.18	4.39
TG(16:0_28:1)	8.98	3.83	2.25	0.70	1.05	5.81	4.27	1.64	0.72	4.57	6.19	14.59	2.06	2.35	2.83	6.51	15.46	9.58	0.75	4.32	1.03	3.02	4.00	11.38	2.51	3.52	6.26	0.64	< LOD	8.12	3.48	< LOD	15.95	4.00	8.70	8.43	8.68	4.93	1.69	5.79	8.11	3.15	3.38	4.01	6.31	6.01	18.05
TG(16:0_28:2)	1.14	0.96	0.28	< LOD	< LOD	0.52	0.47	< LOD	< LOD	0.80	0.70	1.53	0.31	0.47	0.44	0.70	1.51	1.07	< LOD	0.69	0.23	0.46	0.91	1.15	0.48	0.42	0.71	< LOD	< LOD	1.02	0.42	< LOD	1.65	1.04	0.94	1.62	1.82	0.51	0.31	0.65	0.97	0.46	0.55	0.84	1.17	1.21	1.84
TG(16:0_30:2)	1.63	12.42	0.63	< LOD	< LOD	1.17	0.84	< LOD	2.18	6.58	1.67	2.71	0.57	3.41	0.87	1.64	2.27	1.44	0.27	5.24	0.36	0.75	11.25	2.24	1.74	0.79	0.97	1.43	< LOD	1.38	0.99	< LOD	2.75	10.64	1.67	23.43	24.20	1.17	4.17	1.52	2.45	1.53	7.93	11.52	16.84	13.33	2.79
TG(16:0_32:0)	33.84	29.78	10.38	4.20	8.96	37.45	21.62	9.41	7.62	33.44	49.47	89.66	16.57	10.93	45.48	78.31	127.79	82.34	7.45	38.57	3.62	17.45	35.76	94.04	16.48	18.88	35.90	8.42	5.84	39.06	24.27	4.59	86.29	29.88	73.50	77.11	98.69	35.88	11.66	62.77	61.94	33.61	39.22	44.47	63.07	65.85	99.94
TG(16:0_32:1)	34.16	24.63	14.18	4.83	9.51	39.58	21.50	9.70	7.44	24.05	42.15	78.85	16.16	11.22	30.84	53.83	114.59	65.62	6.16	21.96	5.12	19.35	23.02	78.60	14.84	18.72	32.78	8.91	4.83	47.66	25.87	5.04	84.54	22.16	52.63	56.85	64.95	33.58	9.56	48.24	50.20	29.98	20.31	29.78	38.02	34.19	92.88
TG(16:0_32:2)	4.85	5.66	2.32	< LOD	1.81	6.44	3.08	1.46	1.87	4.01	5.65	9.83	2.40	2.89	4.44	7.13	12.41	7.10	0.97	4.23	1.28	3.20	4.89	10.02	2.72	2.97	4.22	2.01	< LOD	7.03	3.93	< LOD	11.30	4.82	7.17	11.57	11.74	4.75	2.36	6.52	9.00	5.45	4.72	7.08	7.60	7.08	11.25
TG(16:0_33:1)	5.22	1.43	2.61	< LOD	1.49	4.64	2.66	1.59	< LOD	2.52	5.12	9.55	2.51	1.19	4.39	6.38	16.58	9.18	1.01	2.88	1.03	3.46	0.93	13.46	1.76	3.08	4.93	1.04	< LOD	7.63	3.29	< LOD	9.63	1.12	6.18	1.69	2.46	4.12	< LOD	5.91	5.09	3.76	1.34	1.16	1.19	1.95	13.72
TG(16:0_34:0)	17.24	11.17	7.24	2.99	5.57	24.28	10.64	6.41	3.88	13.48	18.92	39.76	7.84	5.42	11.78	19.97	56.16	40.51	3.05	14.52	2.57	8.16	11.40	42.72	6.73	8.62	18.78	4.85	2.59	28.88	8.69	2.45	36.93	11.06	26.17	28.97	36.65	20.55	4.51	23.21	19.03	10.33	10.33	13.68	17.09	22.16	47.50
TG(16:0_34:1)	64.04	75.38	33.74	10.70	25.70	102.66	40.35	25.46	27.01	86.13	89.15	161.39	36.23	36.15	57.05	82.15	205.63	126.84	14.09	75.87	11.21	38.81	74.72	173.53	34.00	36.46	68.46	24.87	10.67	106.36	50.46	11.46	164.96	79.42	102.52	206.22	249.43	85.54	25.24	91.41	79.14	53.19	78.59	94.44	131.73	131.11	177.40
TG(16:0_34:2)	12.18	19.29	7.00	3.26	8.19	22.09	8.85	5.55	9.22	20.89	17.66	29.23	8.34	10.16	11.70	17.77	37.24	23.57	3.45	17.14	4.09	9.31	18.39	33.41	8.80	7.88	14.74	9.20	2.58	25.48	10.59	3.06	31.71	18.09	18.43	49.47	54.69	15.52	7.85	19.52	18.38	13.58	23.42	28.76	31.92	35.55	37.65
TG(16:0_34:3)	3.01	2.09	1.58	< LOD	1.68	3.38	1.62	1.10	1.15	2.79	3.30	5.77	1.52	1.61	1.50	2.03	5.52	3.23	0.48	3.12	0.98	1.29	1.47	6.17	2.20	1.75	2.66	1.22	< LOD	5.50	2.33	< LOD	4.73	1.93	3.51	3.17	4.35	2.76	1.01	2.69	3.65	2.36	2.24	2.55	1.92	3.02	6.36
TG(16:0_35:2)	1.95	1.07	1.36	< LOD	1.32	2.03	1.37	0.99	< LOD	1.03	2.03	3.05	1.41	0.89	1.76	2.01	5.68	3.74	0.44	1.33	0.56	2.01	0.37	5.21	1.11	1.18	2.01	< LOD	< LOD	4.39	1.49	< LOD	3.45	< LOD	2.14	< LOD	1.59	1.61	< LOD	2.46	2.49	1.81	0.85	1.14	< LOD	1.10	4.56
TG(16:0_36:2)	23.88	32.65	21.64	6.79	18.07	53.11	15.25	12.59	14.25	34.81	23.25	49.30	12.93	19.51	14.62	18.47	68.74	45.01	5.10	35.81	8.90	18.47	36.37	65.44	14.49	14.84	26.91	13.52	3.26	55.72	13.83	3.93	47.97	38.49	23.30	109.11	123.32	31.38	18.08	39.66	31.94	24.25	45.14	56.19	83.47	85.10	83.40
TG(16:0_36:3)	4.77	17.27	3.48	1.68	3.17	8.50	2.76	1.78	6.47	10.11	5.61	12.09	3.22	8.24	3.03	3.95	13.10	8.46	1.08	13.13	2.97	3.24	16.90	12.96	5.29	2.96	6.15	5.00	0.94	11.24	2.64	1.03	11.14	17.65	5.77	41.16	42.95	6.37	7.30	7.80	7.81	5.04	18.45	22.92	28.71	29.30	15.64
TG(16:0_36:4)	1.53	3.30	1.28	0.73	1.21	2.54	1.30	0.68	1.68	1.83	1.23	3.52	0.94	1.90	0.54	1.21	2.23	1.73	0.42	2.86	1.24	1.05	2.38	3.30	1.62	0.99	1.56	1.34	< LOD	3.02	0.87	< LOD	2.70	2.63	1.46	5.25	5.73	1.49	1.54	1.80	2.25	1.63	2.38	4.00	4.32	4.18	2.93
TG(16:1_28:0)	3.82	1.56	1.27	0.43	0.37	1.78	1.74	0.55	0.47	2.77	3.26	5.96	1.35	1.24	1.18	2.89	4.48	2.55	< LOD	1.31	0.26	0.94	0.84	3.44	1.39	1.06	1.45	0.63	0.35	3.91	1.90	0.39	6.90	1.43	4.65	3.16	3.66	2.11	0.59	3.22	4.32	1.32	1.08	1.66	1.36	2.03	4.38
TG(16:1_30:1)	1.81	1.99	1.28	< LOD	0.71	2.53	1.31	< LOD	0.69	2.34	2.37	3.15	0.74	1.34	1.72	2.75	3.77	2.21	< LOD	1.14	0.58	1.32	1.19	3.77	1.30	0.69	1.68	0.78	< LOD	3.32	1.68	< LOD	4.60	1.26	2.67	2.54	3.08	1.45	0.77	2.00	2.77	1.60	1.00	1.39	1.09	1.48	2.70
TG(16:1_32:0)	6.51	2.62	2.96	1.44	2.44	8.69	4.52	2.52	1.48	4.07	10.43	15.77	4.02	1.71	8.90	15.65	21.31	12.32	1.67	2.97	1.37	3.87	1.55	18.99	2.67	3.77	6.83	2.10	1.68	11.64	6.57	1.56	19.31	1.68	13.14	3.41	4.66	8.07	1.14	10.11	11.29	7.64	2.04	3.37	1.52	3.21	15.98
TG(16:1_32:1)	4.87	2.15	3.03	1.30	2.80	6.50	3.45	1.77	< LOD	2.49	5.95	8.88	2.72	1.40	5.18	8.70	14.14	7.67	1.46	1.34	1.38	3.83	1.14	11.63	1.62	3.21	4.40	1.55	< LOD	9.10	4.78	1.54	11.76	< LOD	6.22	2.47	2.33	5.06	< LOD	7.16	7.81	5.56	1.37	2.14	< LOD	1.72	10.14
TG(16:1_34:1)	8.88	4.80	7.30	2.86	7.56	20.94	7.35	5.54	2.96	6.81	14.03	20.97	6.69	3.74	11.60	16.39	32.03	19.27	3.36	5.98	3.57	9.91	3.17	30.75	5.57	7.41	12.33	5.41	2.46	22.96	9.79	3.18	26.89	3.46	15.67	7.34	9.38	12.93	1.87	14.91	15.22	11.76	3.79	6.37	3.64	6.23	29.52
TG(16:1_34:2)	1.74	1.74	1.55	< LOD	1.86	3.88	1.50	< LOD	1.59	1.65	2.68	4.02	1.63	1.45	2.25	3.01	4.58	2.86	0.51	0.99	1.25	1.79	0.76	4.76	1.14	1.26	2.11	1.92	< LOD	4.90	1.89	< LOD	5.02	< LOD	2.75	1.73	1.93	2.67	< LOD	3.28	2.57	3.26	1.49	2.26	1.48	1.50	5.24
TG(16:1_36:1)	2.26	0.72	1.36	< LOD	1.79	4.45	1.40	1.22	0.81	1.02	2.39	3.59	0.82	0.76	1.32	1.53	5.47	3.35	0.27	1.00	0.55	1.35	0.66	4.86	0.59	1.29	1.89	0.88	< LOD	5.65	1.09	< LOD	4.00	< LOD	1.65	0.94	1.20	2.37	< LOD	2.13	1.18	1.27	0.45	0.87	0.60	0.94	6.14
TG(16:1_36:2)	2.69	2.12	2.49	1.54	3.50	8.30	2.04	2.20	1.41	2.47	3.84	6.01	1.89	1.96	1.89	2.54	7.02	4.72	0.85	2.21	1.74	3.07	1.35	8.05	1.76	2.42	3.79	2.48	0.73	10.13	1.99	0.89	6.39	1.20	2.70	3.26	3.66	4.05	1.11	3.65	3.36	2.77	1.27	2.35	1.54	2.17	8.81
TG(16:1_36:3)	0.49	0.76	0.68	< LOD	0.90	1.14	0.59	0.37	0.64	0.72	0.83	1.18	0.74	0.64	0.41	0.74	1.05	0.88	0.18	0.77	0.72	0.61	0.30	1.61	0.51	0.39	0.81	0.65	< LOD	1.61	0.66	0.52	1.08	0.50	0.59	1.04	1.17	0.96	0.47	0.88	1.00	0.88	0.58	1.06	0.76	0.85	2.27
TG(17:0_34:1)	3.03	0.84	1.76	< LOD	1.32	4.40	2.26	1.55	< LOD	1.17	2.71	5.14	1.42	0.75	2.11	2.99	9.94	6.24	0.70	1.83	0.96	2.24	0.66	9.18	1.40	2.34	3.69	0.84	< LOD	6.35	1.58	< LOD	5.68	0.70	2.85	0.89	1.73	2.82	< LOD	3.40	3.17	2.23	0.93	1.05	0.83	1.30	10.65
TG(18:0_30:0)	6.14	1.71	1.42	< LOD	< LOD	4.47	3.34	1.54	< LOD	1.84	3.59	9.44	1.83	< LOD	1.80	4.03	14.41	11.23	< LOD	1.85	< LOD	1.31	1.39	8.83	1.38	1.82	4.19	< LOD	< LOD	6.58	1.77	< LOD	9.77	1.73	6.20	4.06	4.33	4.39	< LOD	6.57	5.33	2.11	1.50	1.70	3.00	2.55	13.45
TG(18:0_30:1)	2.64	2.64	< LOD	< LOD	0.82	2.21	1.21	< LOD	< LOD	2.09	1.60	3.34	< LOD	1.17	1.08	2.02	7.44	5.15	< LOD	3.87	0.54	1.06	4.81	5.58	1.83	1.09	2.98	< LOD	< LOD	3.24	< LOD	< LOD	4.26	3.18	1.98	7.02	8.53	1.79	1.53	2.40	2.15	0.85	2.61	3.57	5.68	4.94	6.35
TG(18:0_32:0)	10.21	2.92	3.54	1.51	2.66	13.18	5.84	3.23	< LOD	4.60	8.44	17.85	3.77	1.60	4.96	8.69	30.54	21.12	0.95	5.13	1.32	3.56	3.58	22.73	2.71	3.46	11.35	1.54	< LOD	14.36	3.80	< LOD	19.05	3.41	13.12	7.53	9.80	10.24	1.89	14.36	9.31	4.89	3.63	3.47	5.38	6.07	28.45
TG(18:0_32:1)	9.59	2.77	3.40	1.50	2.90	10.82	5.19	3.20	< LOD	2.74	5.84	14.22	3.06	1.50	3.57	6.01	30.87	20.35	1.00	3.11	1.89	4.36	2.32	21.71	2.40	5.00	10.50	1.64	< LOD	14.35	2.79	< LOD	16.70	2.47	7.59	5.04	6.66	9.12	1.38	10.52	5.89	3.36	1.75	3.24	3.60	4.46	24.31
TG(18:0_34:2)	3.90	2.44	1.80	0.72	2.35	6.09	2.26	1.96	1.44	3.35	3.22	7.23	1.37	1.27	1.62	1.92	8.59	6.23	< LOD	3.44	1.33	1.71	1.96	6.81	1.78	1.79	4.36	1.28	< LOD	9.39	1.32	0.63	8.29	2.38	3.00	5.97	6.41	4.41	1.56	4.80	2.55	1.95	2.48	4.02	3.76	3.50	12.62
TG(18:0_34:3)	0.69	0.39	< LOD	0.40	0.31	0.98	0.58	0.35	< LOD	0.62	0.57	0.79	0.45	< LOD	0.20	0.34	1.38	1.10	< LOD	0.44	0.23	< LOD	0.26	1.69	0.53	0.53	0.91	0.39	< LOD	1.68	0.31	0.27	0.87	< LOD	0.67	0.55	0.53	0.84	0.51	1.07	0.85	0.35	0.63	0.43	0.75	0.47	2.01
TG(18:0_36:2)	9.21	8.70	3.96	< LOD	5.15	14.37	5.94	6.25	< LOD	6.09	5.82	13.32	< LOD	4.72	< LOD	< LOD	28.00	20.27	< LOD	8.09	< LOD	7.05	12.29	26.28	< LOD	4.68	14.59	< LOD	< LOD	19.28	< LOD	< LOD	15.38	4.18	4.99	11.10	16.65	8.93	< LOD	7.99	5.85	5.15	< LOD	5.16	4.92	7.33	32.75
TG(18:0_36:3)	1.89	3.33	0.84	< LOD	1.59	2.46	0.93	1.49	1.30	2.30	0.96	2.89	1.33	1.64	0.22	0.47	2.81	2.01	0.40	2.81	0.49	0.41	2.29	3.10	1.27	0.75	2.19	0.95	< LOD	4.91	< LOD	< LOD	2.64	3.35	1.10	6.97	8.20	1.84	1.50	1.54	1.81	< LOD	2.83	3.90	3.26	3.61	4.70
TG(18:1_26:0)	16.75	22.24	4.62	1.31	1.53	8.83	7.29	2.77	2.67	16.03	8.96	22.52	3.22	7.83	2.25	4.15	17.45	11.12	0.68	10.44	1.22	3.30	17.02	12.72	5.52	4.05	8.04	2.45	0.43	14.76	5.21	0.40	23.87	19.95	11.45	49.35	50.88	7.25	7.09	9.50	9.74	4.04	10.94	13.57	27.72	20.41	25.58
TG(18:1_28:1)	7.11	4.68	2.63	0.99	1.45	5.89	3.65	1.80	0.78	3.93	4.51	9.59	1.46	2.81	1.38	2.95	9.39	6.28	0.54	3.61	1.35	2.72	3.54	8.99	1.96	2.22	4.79	0.73	< LOD	9.62	2.42	< LOD	11.14	3.70	3.73	7.73	8.86	3.86	1.54	4.40	4.95	2.23	2.42	3.88	5.52	4.63	13.96
TG(18:1_30:0)	33.68	32.81	14.14	4.27	8.51	34.70	19.62	8.38	5.86	26.71	33.15	65.82	14.06	14.14	19.73	38.36	102.28	61.41	4.31	26.80	4.92	16.67	32.96	70.11	17.33	17.97	31.84	7.65	3.30	44.59	22.33	2.88	74.05	29.95	40.33	73.20	81.65	29.89	11.91	41.00	44.12	22.73	22.45	32.50	45.72	42.80	76.80
TG(18:1_30:1)	13.82	55.70	7.29	2.10	3.42	15.46	6.88	3.37	8.61	31.86	11.93	19.76	4.69	20.81	6.22	10.72	25.61	15.88	1.22	31.50	3.13	7.38	57.25	23.23	11.27	6.79	9.82	6.07	< LOD	20.08	6.99	1.68	22.85	45.38	10.37	115.18	126.25	9.53	20.83	11.52	15.31	7.24	29.38	46.48	75.69	56.22	26.41
TG(18:1_30:2)	1.11	11.11	< LOD	< LOD	< LOD	1.11	0.84	< LOD	1.83	5.08	0.99	1.68	< LOD	3.25	0.46	0.82	1.71	1.32	0.23	5.92	0.61	0.78	12.18	2.00	1.71	0.73	0.92	1.41	< LOD	1.60	< LOD	< LOD	2.06	8.47	0.97	18.62	21.18	< LOD	4.77	0.93	1.53	< LOD	6.40	9.53	12.81	10.25	2.29
TG(18:1_32:0)	48.28	45.39	27.96	7.37	17.68	75.46	30.56	18.46	14.42	47.92	56.23	108.82	23.12	23.39	38.01	63.32	163.21	101.85	9.82	51.65	9.26	28.00	46.06	123.79	24.13	29.21	54.67	14.93	5.49	83.32	36.47	7.59	108.58	42.56	62.59	120.15	152.36	54.10	18.65	66.02	61.11	37.07	49.70	62.25	83.25	81.28	139.14
TG(18:1_32:1)	40.03	32.18	27.33	8.81	18.55	66.17	22.83	14.50	8.60	28.18	39.31	70.87	18.10	20.39	20.39	30.13	96.70	59.05	5.29	25.18	10.76	24.17	24.10	83.69	16.66	20.68	36.34	10.86	4.22	81.82	26.23	5.06	77.56	25.84	36.18	64.91	77.38	40.80	12.11	42.80	38.47	24.39	19.27	27.15	34.51	34.37	90.07
TG(18:1_32:2)	3.82	5.52	3.05	1.33	2.55	7.57	2.55	1.51	1.94	4.08	4.58	6.83	2.09	3.16	2.48	3.58	9.12	5.86	0.79	3.71	1.99	3.37	4.84	9.04	2.48	2.45	3.80	1.63	< LOD	9.29	2.49	< LOD	7.99	4.72	3.28	10.58	11.72	3.99	2.62	3.69	4.54	3.03	3.76	5.99	6.59	5.34	10.01
TG(18:1_33:0)	4.74	1.03	2.46	0.87	2.03	6.60	2.42	1.62	< LOD	1.37	3.20	7.37	1.61	1.10	2.63	3.81	13.79	9.59	0.82	2.41	1.11	3.86	0.49	12.45	1.69	2.36	5.35	0.97	< LOD	9.18	1.77	< LOD	7.26	0.95	3.29	1.40	1.64	3.35	< LOD	3.84	4.09	2.40	< LOD	0.99	1.16	1.32	14.51
TG(18:1_33:1)	5.56	1.30	4.84	1.51	2.65	8.78	3.30	2.69	< LOD	2.30	5.31	8.82	2.59	1.39	2.78	3.33	12.61	7.50	0.88	2.16	1.81	3.90	0.77	12.26	1.79	3.07	4.93	1.24	0.92	14.59	3.50	0.91	9.10	0.95	4.00	2.24	2.88	5.09	< LOD	5.43	5.28	3.56	1.35	1.35	1.54	1.57	17.63
TG(18:1_34:1)	62.31	86.12	65.29	19.29	53.17	158.40	41.60	36.63	30.67	92.38	71.26	135.63	33.16	55.15	44.67	55.24	196.50	138.48	14.96	90.33	30.11	61.59	86.72	203.56	41.16	48.50	88.24	29.32	7.88	177.70	51.96	11.98	144.27	80.43	61.70	235.79	293.07	90.65	38.11	80.61	71.66	53.06	84.97	104.17	142.47	138.23	203.94
TG(18:1_34:2)	12.09	22.48	9.51	4.84	10.11	27.79	6.89	5.93	9.12	18.77	12.91	21.98	6.59	14.16	7.04	8.76	28.33	17.15	2.61	17.19	6.71	9.92	18.64	30.13	8.96	6.98	14.57	10.31	1.93	35.35	7.95	2.46	22.12	20.33	10.68	48.10	60.36	14.52	9.93	14.26	14.16	10.87	20.60	27.36	31.39	30.07	39.77
TG(18:1_34:3)	2.11	1.77	1.64	0.65	1.42	3.63	0.97	0.94	0.82	2.09	2.08	3.92	1.32	1.46	0.78	1.19	3.19	2.39	0.44	2.51	1.75	1.36	1.33	4.22	1.49	1.15	1.82	1.48	< LOD	5.98	1.13	0.40	3.60	1.39	1.48	2.60	4.06	2.46	0.72	2.33	2.54	1.75	1.69	2.53	1.91	2.53	5.78
TG(18:1_35:2)	1.08	0.39	1.30	0.46	0.93	2.83	0.63	0.73	0.32	0.50	0.87	1.78	0.61	0.78	0.62	0.66	3.20	2.25	0.25	1.02	0.84	1.21	0.33	3.45	0.80	0.70	1.36	< LOD	< LOD	3.82	0.78	< LOD	1.80	0.44	0.77	0.54	1.16	0.79	< LOD	1.09	0.90	0.71	0.32	0.65	0.51	0.55	4.13
TG(18:1_36:1)	15.01	12.12	8.10	< LOD	9.05	28.04	6.30	7.52	4.28	14.68	9.77	18.67	5.95	8.47	< LOD	< LOD	29.25	21.95	< LOD	8.04	1.42	6.44	8.02	29.45	3.11	7.11	15.58	< LOD	< LOD	35.06	5.46	< LOD	22.90	11.34	7.42	26.56	40.08	12.64	< LOD	11.90	7.16	< LOD	11.02	12.15	16.74	19.09	47.79
TG(18:1_36:2)	17.01	31.43	15.59	4.79	15.57	44.64	9.57	9.36	10.29	29.26	12.11	25.14	7.47	19.15	4.34	5.63	32.94	24.88	< LOD	26.41	7.95	15.75	25.68	41.00	9.35	8.56	15.85	9.23	< LOD	48.09	6.41	2.00	30.96	26.13	7.79	71.61	93.44	15.21	16.71	15.35	11.65	10.37	25.08	31.84	47.23	45.56	64.45
TG(18:1_36:3)	4.90	15.70	3.80	< LOD	2.82	8.64	2.75	1.84	5.23	9.94	4.22	7.75	2.64	9.70	1.40	2.15	8.10	5.74	0.54	10.99	3.57	3.08	14.45	9.18	4.96	2.57	4.49	3.31	< LOD	10.02	1.90	< LOD	6.75	12.78	2.81	31.53	41.02	4.49	6.64	4.80	4.09	2.55	7.86	14.20	20.98	19.83	12.75
TG(18:1_36:4)	0.95	3.03	0.96	0.42	0.86	2.72	0.79	< LOD	0.93	1.28	1.21	1.87	0.65	1.85	0.67	0.42	1.14	1.06	< LOD	1.95	0.92	0.87	1.48	2.08	1.31	0.73	1.39	0.91	< LOD	2.77	0.86	< LOD	1.89	1.87	0.79	4.09	4.93	0.91	1.28	1.32	1.10	0.69	2.26	2.45	3.01	2.69	3.33
TG(18:1_38:5)	0.39	0.33	0.51	< LOD	0.29	0.37	0.30	< LOD	< LOD	0.44	0.40	0.73	0.46	0.35	< LOD	0.27	1.11	0.62	< LOD	0.66	0.40	0.23	0.41	1.15	0.53	0.33	0.34	0.30	0.35	0.73	< LOD	< LOD	0.81	< LOD	< LOD	0.70	0.72	0.41	0.64	< LOD	0.93	0.85	0.81	0.92	0.68	0.71	1.03
TG(18:2_28:0)	2.24	13.25	0.68	< LOD	< LOD	1.13	0.88	< LOD	2.22	7.25	1.64	3.29	0.64	4.37	0.63	1.10	3.25	2.32	0.24	7.65	0.38	0.45	14.18	2.31	3.01	0.88	1.70	1.80	< LOD	2.12	0.88	< LOD	3.49	11.68	1.80	25.41	27.94	1.29	5.09	1.53	2.19	0.97	8.22	12.17	18.91	15.01	4.12
TG(18:2_30:0)	3.85	8.02	1.76	< LOD	< LOD	2.79	2.09	< LOD	2.12	5.38	3.34	6.96	1.79	3.07	1.73	2.64	7.59	4.41	0.36	5.01	0.63	1.09	7.62	5.32	2.57	1.50	2.37	1.60	< LOD	4.23	1.80	< LOD	6.29	6.75	4.40	16.01	16.99	3.00	3.25	4.21	4.82	2.00	5.74	8.95	10.85	9.44	7.21
TG(18:2_30:1)	1.73	13.60	< LOD	< LOD	< LOD	1.44	< LOD	< LOD	2.34	5.93	1.66	2.07	< LOD	4.91	0.55	0.91	2.14	1.60	0.15	6.60	0.70	0.68	12.48	2.05	2.03	0.75	0.97	1.78	< LOD	2.38	< LOD	< LOD	2.03	10.15	1.48	22.12	25.20	1.06	4.92	1.25	1.61	0.86	5.99	9.79	14.92	10.62	2.33
TG(18:2_32:0)	5.72	12.08	2.28	1.42	2.30	7.22	3.54	1.73	4.50	9.78	6.60	12.83	2.84	5.91	3.04	5.44	13.52	8.31	1.01	10.42	1.56	2.56	10.95	11.44	4.22	2.63	5.29	3.87	< LOD	8.74	3.63	< LOD	11.05	13.57	8.09	27.78	33.03	6.38	5.04	6.82	7.23	4.68	13.75	16.10	19.46	19.39	16.26
TG(18:2_32:1)	3.68	6.33	2.04	< LOD	1.75	4.01	2.09	1.21	2.24	4.75	3.35	5.88	1.74	3.80	1.60	2.63	6.59	4.72	0.38	3.96	1.60	1.90	5.14	6.31	2.37	1.97	3.30	2.09	< LOD	6.82	2.13	< LOD	6.49	5.05	3.53	10.94	13.48	3.89	2.27	3.24	3.53	2.15	4.27	5.85	6.53	5.73	7.82
TG(18:2_34:0)	2.97	3.38	1.29	0.85	1.36	3.80	1.54	1.18	1.73	3.42	1.87	4.79	1.43	1.96	1.17	1.28	6.18	4.21	0.45	3.30	0.75	1.05	3.18	4.88	1.60	0.82	2.97	1.58	< LOD	4.84	1.07	< LOD	4.42	3.57	2.29	9.17	11.79	3.04	1.98	3.03	2.06	1.26	3.25	4.46	5.12	5.37	7.91
TG(18:2_34:1)	5.64	18.76	4.59	2.31	3.86	10.92	3.86	2.65	7.91	14.10	6.98	13.25	3.47	10.80	2.93	3.55	14.73	10.26	1.11	14.57	3.46	4.32	17.69	14.55	5.82	3.47	7.47	6.01	0.89	16.24	3.46	1.15	11.73	16.86	6.37	43.82	53.80	7.96	8.88	6.57	6.04	4.67	16.50	22.83	29.53	24.88	19.03
TG(18:2_34:2)	1.21	4.58	1.23	< LOD	0.95	1.96	0.84	< LOD	2.43	2.39	1.64	2.84	0.79	3.01	0.59	0.89	2.22	1.47	< LOD	2.93	1.07	0.96	3.18	2.67	1.70	0.60	1.38	1.83	< LOD	3.43	< LOD	< LOD	1.90	4.13	1.20	8.62	9.01	1.37	2.46	1.25	1.64	1.33	3.80	6.35	5.35	4.58	3.39
TG(18:2_36:1)	1.26	3.22	< LOD	< LOD	1.04	2.93	1.25	0.88	1.86	2.32	0.82	3.02	0.88	1.49	0.48	0.41	3.54	1.85	0.43	3.01	1.26	0.69	2.34	2.43	1.01	0.92	1.92	0.84	< LOD	3.77	< LOD	< LOD	2.75	2.77	1.01	6.07	6.18	1.91	1.92	1.51	1.16	< LOD	2.75	4.05	4.71	3.70	4.51
TG(18:2_36:2)	2.33	10.30	1.91	< LOD	1.98	4.22	1.46	< LOD	2.96	5.53	1.50	3.42	1.39	5.56	0.71	0.74	3.27	2.89	0.21	4.72	1.67	1.45	6.18	3.88	1.79	0.85	1.46	2.48	< LOD	5.59	< LOD	< LOD	2.93	7.50	1.37	16.50	22.09	1.78	4.18	2.52	1.21	1.57	6.09	7.23	10.14	9.15	6.74
TG(18:3_34:1)	1.48	1.29	1.54	0.51	0.67	2.10	1.10	0.52	0.62	1.45	1.29	2.70	0.97	1.21	0.48	0.52	2.53	1.64	< LOD	1.74	0.85	1.10	0.97	3.44	1.10	0.84	1.58	0.83	< LOD	3.97	1.07	< LOD	2.31	1.29	1.26	2.09	2.96	1.74	0.75	1.84	1.93	1.11	1.52	1.72	1.39	2.14	4.21
TG(18:3_36:2)	0.46	0.33	0.63	0.21	0.32	0.99	0.37	0.22	0.36	0.56	0.24	0.74	0.46	0.51	< LOD	< LOD	0.47	0.33	0.19	0.49	0.40	0.25	0.32	0.74	0.32	0.24	0.28	0.20	< LOD	1.50	0.22	< LOD	0.73	0.29	0.30	0.78	1.26	0.38	0.46	0.49	0.47	0.35	0.51	0.37	0.86	1.01	1.11
TG(20:4_34:1)	0.42	0.38	0.74	< LOD	0.34	0.90	0.63	0.45	0.37	0.60	0.94	1.14	0.46	0.51	0.45	0.62	1.33	1.14	0.14	0.66	0.35	0.47	0.40	1.75	0.62	0.32	0.59	0.72	< LOD	0.78	0.42	< LOD	0.91	0.37	0.42	0.99	1.05	0.75	0.55	0.73	1.12	1.14	1.15	1.12	0.88	1.18	1.51
Hex3Cer(d18:1/16:0)	< LOD	0.38	< LOD	0.32	0.35	0.17	0.19	0.22	0.32	0.22	0.35	0.22	0.25	0.19	0.18	0.14	0.17	0.14	0.06	0.30	0.26	0.14	0.27	0.14	0.38	0.20	0.35	0.20	0.18	< LOD	< LOD	0.18	0.25	0.26	< LOD	0.47	0.38	0.39	0.31	0.39	< LOD	< LOD	0.31	0.33	0.41	0.58	0.32
Choline	5.60	12.91	13.83	10.57	22.31	6.72	15.24	11.39	13.83	13.32	15.24	11.12	18.88	9.61	11.48	21.68	7.88	9.86	20.73	16.10	19.29	22.00	16.73	13.16	15.52	20.07	12.43	30.40	7.43	9.52	15.72	12.82	6.98	7.74	8.28	8.47	12.97	16.62	31.98	29.22	8.21	11.87	13.91	22.30	14.29	11.86	14.92
