# Supplementary material for: A Pilot Study on Across-Generation Impacts of Maternal Heat Stress on Blood Metabolites of Female Holstein Dairy Calves
Source: Metabolites. 2023 Mar 29;13(4):494. doi: 10.3390/metabo13040494 (PMC10141042; doi:10.3390/metabo13040494)
Supplement: Supplementary file 1 [file metabolites-13-00494-s001.zip › Table S2.pdf]

Supplementary Materials:

Table S2: Effects of fixed effects in models 1-3 on metabolite concentrations of calves. The table includes log transformed Least-squares means (LSMeans) with corresponding SE, number of observations (n) and P-value, adjusted with Bonferroni correction. *$p < 0.05$; ** $p < 0.01$; *** $p < 0.001$

Metabolite	Model	Fixed effect	Effect class	LSMeans (log transformed)	SE	*n*	*P*-value
PC aa C34:1	1	mTHI-class	mTHI >= 60	1.8021	0.03793	14	**
			mTHI < 60	1.9352	0.02471	33	
PC aa C38:3		mTHI-class	mTHI >= 60	0.9060	0.03208	14	***
			mTHI < 60	1.0612	0.02090	33	
PC aa C38:4		mTHI-class	mTHI >= 60	1.0458	0.03927	14	**
			mTHI < 60	1.1837	0.02558	33	
PC ae C38:3		mTHI-class	mTHI >= 60	-0.1098	0.03757	14	***
			mTHI < 60	0.05282	0.02447	33	
PC ae C40:4		mTHI-class	mTHI >= 60	-0.6114	0.03358	14	***
			mTHI < 60	-0.4690	0.02187	33	
CE(20:3)		mTHI-class	mTHI >= 60	0.3059	0.03911	14	***
			mTHI < 60	0.5256	0.02548	33	
CE(20:4)		mTHI-class	mTHI >= 60	1.0951	0.04316	14	***
			mTHI < 60	1.2939	0.02811	33	
CE(22:5)		mTHI-class	mTHI >= 60	-0.4238	0.03971	13	*
			mTHI < 60	-0.2981	0.02492	33	
H1		mTHI-class	mTHI >= 60	3.7963	0.02755	14	**
			mTHI < 60	3.8943	0.01795	33	
PC aa C38:1	2	mTHI-class	mTHI >= 60	-0.8397	0.1256	14	**
			mTHI < 60	-0.3794	0.1001	33	
		calving condition	no birth assistance	-0.5899	0.07153	35	n.s.
			minor birth assistance/dystocia	-0.6292	0.1667	6	
P-Cresol-SO4		mTHI-class	mTHI >= 60	-0.3700	0.1428	14	*
			mTHI < 60	-0.02230	0.1138	33	
		calving condition	no birth assistance	-0.1092	0.08133	35	n.s.
			minor birth assistance/dystocia	-0.2832	0.1895	6	
SM C18:0	3	mTHI-class	mTHI >= 60	0.4269	0.03276	14	***
			mTHI < 60	0.5985	0.02061	33	
		age at blood sampling (d)	1	0.3809	0.04402	8	***
			2 to 3	0.4519	0.03292	13	
			4 to 5	0.5875	0.03292	13	
			6 to 7	0.6304	0.03423	13	
SM C18:1		mTHI-class	mTHI >= 60	0.003073	0.03375	14	***
			mTHI < 60	0.1659	0.02123	33	
		age at blood sampling (d)	1	-0.05998	0.04535	8	***
			2 to 3	0.02738	0.03391	13	
			4 to 5	0.1536	0.03391	13	
			6 to 7	0.2168	0.03526	13	

SM C24:1	mTHI-class	mTHI >= 60	0.5739	0.03056	14	***
		mTHI < 60	0.7165	0.01923	33	
	age at blood sampling (d)	1	0.5202	0.04106	8	***
		2 to 3	0.6015	0.03071	13	
		4 to 5	0.6841	0.03071	13	
		6 to 7	0.7748	0.03193	13	
PC ae C30:2	mTHI-class	mTHI >= 60	-1.5266	0.04253	14	***
		mTHI < 60	-1.3481	0.02676	33	
	age at blood sampling (d)	1	-1.5965	0.05715	8	**
		2 to 3	-1.4206	0.04274	13	
		4 to 5	-1.3613	0.04274	13	
		6 to 7	-1.3709	0.04444	13	
